# Assessing the integration of social marketing principles in ivory demand management interventions in China and Southeast Asia

**DOI:** 10.1111/cobi.70191

**Published:** 2025-12-16

**Authors:** Molly R. C. Brown, Victoria K. Wells, Colin M. Beale

**Affiliations:** ^1^ Leverhulme Centre for Anthropocene Biodiversity University of York York UK; ^2^ School for Business and Society University of York York UK; ^3^ Department of Biology University of York York UK

**Keywords:** behavior change, China, consumer demand, elephant ivory, social marketing, cambio conductual, China, demanda del consumidor, marfil, mercadotecnia social, 象牙, 社会营销, 行为改变, 消费者需求, 中国

## Abstract

Consumer demand for ivory perpetuates the unsustainable and illegal killing of African elephants and other wildlife species. Interventions that aim to change consumer behavior are increasingly recognized as a crucial element of demand management. However, poor design and implementation have limited their effectiveness. We evaluated how ivory demand‐management interventions in China and neighboring Southeast Asian countries align with best practices from the behavioral field of social marketing. Through a literature review, we identified 55 interventions conducted from 2008 to 2022. We used 2 social marketing frameworks to assess each intervention's capacity to influence behavior. We conducted semistructured interviews with 5 intervention practitioners to provide contextual grounding for our review findings. From 2018 to 2022, social marketing principles were more frequently applied and interventions were of a higher quality (*n* = 26) than interventions conducted from 2008 to 2017, reflecting a growing adoption of consumer‐insight‐driven strategies. Since 2018, 7 interventions applied no social marketing principles, and 9 interventions, to varying degrees, included monitoring and evaluation. Although 13 interventions contained some theoretical considerations, these were often vague and superficial. Despite identifying a shift from experiential practices to evidence‐based approaches over time, the shift was largely restricted to communications‐based social and behavior change approaches. This left the wide range of social marketing approaches underused. Ivory demand management must improve the breadth and depth of social marketing used to contribute to long‐term elephant conservation. We suggest all consumer approaches tackling demand for wildlife meaningfully consider integrating behavioral theories in intervention design, undertake primary or secondary research to enable evidence‐led decision‐making, conduct systematic monitoring for evidence‐based learning and adaptation, and use impact and process evaluation methods to understand the mechanisms and magnitude of behavioral change following interventions.

## Abstract (es)

Evaluación de la integración de los principios del mercadotecnia social en las intervenciones de manejo de la demanda de marfil en China y el sudeste asiático

**Resumen:**

La demanda de marfil por parte de los consumidores perpetúa la matanza insostenible e ilegal de elefantes africanos y otras especies silvestres. Las intervenciones destinadas a cambiar el comportamiento de los consumidores se reconocen cada vez más como un elemento crucial del manejo de la demanda. Sin embargo, su eficacia se ha visto limitada por un diseño y una aplicación deficientes. Evaluamos cómo las intervenciones de manejo de la demanda de marfil en China y los países vecinos del sudeste asiático se ajustan a las mejores prácticas del campo de la mercadotecnia social conductual. A través de una revisión bibliográfica, identificamos 55 intervenciones realizadas entre 2008 y 2022. Utilizamos dos marcos de mercadotecnia social para evaluar la capacidad de cada intervención para influir en el comportamiento. Realizamos entrevistas semiestructuradas a cinco profesionales de la intervención para proporcionar una base contextual a los resultados de nuestra revisión. Entre 2018 y 2022, los principios de la mercadotecnia social se aplicaron con mayor frecuencia y las intervenciones fueron de mayor calidad (n = 26) que las realizadas entre 2008 y 2017, lo que refleja una creciente adopción de estrategias basadas en el conocimiento del consumidor. Desde 2018, siete intervenciones no aplicaron principios de mercadotecnia social y nueve intervenciones, en diversos grados, incluyeron seguimiento y evaluación. Si bien trece intervenciones contenían algunas consideraciones teóricas, estas solían ser vagas y superficiales. A pesar de que se identificó un cambio de las prácticas empíricas a los enfoques basados en la evidencia a lo largo del tiempo, dicho cambio se limitó en gran medida a los enfoques de cambio social y conductual basados en la comunicación. Esto dejó sin aprovechar la amplia gama de enfoques de mercadotecnia social. La gestión de la demanda de marfil debe mejorar la amplitud y la profundidad de la mercadotecnia social utilizada para contribuir a la conservación a largo plazo de los elefantes. Sugerimos que todos los enfoques de consumo que aborden la demanda de vida silvestre consideren de manera significativa la integración de teorías conductuales en el diseño de las intervenciones, realicen investigaciones primarias o secundarias que permitan la toma de decisiones basadas en la evidencia, lleven a cabo un seguimiento sistemático para el aprendizaje y la adaptación basados en la evidencia, y utilicen métodos de evaluación del impacto y del proceso para comprender los mecanismos y la magnitud del cambio de comportamiento tras las intervenciones.

## 中国及东南亚地区象牙需求管理干预中社会营销原则的应用评估

消费者对象牙的持续需求, 推动了对非洲象及其他野生动物的非法和不可持续猎杀。旨在改变消费者行为的干预策略正日益被视为需求管理的关键组成部分。然而, 由于设计与实施不当, 此类干预策略的成效有限。本研究评估了中国及周边东南亚国家对象牙需求管理的干预, 在多大程度上符合社会营销领域的行为学最佳实践。通过文献综述, 我们共识别出2008年至2022年间实施的55项干预策略。研究采用了两个社会营销框架来评估每项干预策略对行为的影响潜力。此外, 我们与五位干预策略实践者进行了半结构化访谈, 以此为文献综述提供情境化支撑。 结果显示, 2018年至2022年期间, 社会营销原则的应用更为普遍, 干预质量也显著提高(*n* = 26), 反映出以消费者洞察为驱动的策略正逐步被采纳。自2018年以来, 有7项干预策略未应用任何社会营销原则, 而9项干预策略在不同程度上纳入了监测与评估。尽管有13项干预策略在一定程度上考虑了理论因素, 但多显模糊且流于表面。研究发现, 干预实践正逐步从经验导向转向循证导向, 然而这一转变主要局限于基于传播的社会与行为改变方法, 导致社会营销的潜能仍未得到充分发挥。象牙需求管理亟需更广泛深入地应用社会营销原则, 以助力长期的象类保护。我们建议, 所有针对野生动物需求的消费者干预, 应在设计阶段切实融入行为学理论, 开展初级或次级研究以支持循证决策, 同时建立系统性监测机制, 以实现基于证据的学习与适应, 并通过成效评估与过程评估深入理解干预后的行为变化机制与幅度。

## INTRODUCTION

Human overexploitation of wildlife is a major driver of biodiversity decline (IPBES, 2019). The illegal wildlife trade (IWT) contributes to this decline by capitalizing on consumer demand for wildlife products, such as elephant ivory, through the unlawful buying or selling of harvested wild species (t Sas‐Rolfes et al., 2019). In China and some countries in Southeast Asia, including Japan and Thailand, the market demand for ivory has remained influential in determining African elephant (*Loxodonta africana*, *L. cyclotis*) poaching rates over the past 2 decades (Gao & Clark, 2014; Thomas‐Walters et al., 2023).

In the early 2000s, China's economic and traditional arts investment boom contributed heavily to a peak in the illegal killing of African elephants from 2010 to 2012, causing some populations to decrease by around 60% (Brennan & Kalsi, 2015; Hauenstein et al., 2019). Since 2012, the Chinese government has aimed to protect biodiversity through the Ecological Civilization program (Zhou et al., 2018), which included implementing a domestic ivory ban in late 2017 (China State Council, 2016, 2024; Wang & Jiang, 2018). However, ivory seizures continue to occur across China and Southeast Asia, such as in 2019 when 3 of the largest recorded seizures took place in China (7.5 t), Singapore (8.8 t), and Vietnam (9.1 t) (UNODC, 2024). From 2010 to 2023, efforts to tackle IWT were dominated by enforcement‐driven initiatives, leaving only 5% of global investment for demand reduction and behavior change interventions (World Bank Group, 2025). The use of one‐size‐fits‐all messaging highlights the prevalence of ungrounded environmentalism (Vu, 2023), where historical racist stereotypes misrepresent cultural norms (Margulies et al., 2019). The lack of robust impact evaluation further weakens overall understanding of the effects of demand management on ivory consumer behavior (Greenfield & Veríssimo, 2018).

Drivers of ivory demand are not homogeneous (Liang et al., 2020) and need deep cultural understanding to be addressed ethically and effectively (Thomas‐Walters, Veríssimo, et al., 2020). In China, for example, ivory is often associated with social and cultural norms that assign ivory high social capital value (Gao & Clark, 2014). These norms are exemplified in gifting practices (and sometimes bribery) and adhere to Chinese cultural concepts of Confucian virtuosity, such as benevolence, *rén* (仁), and propriety, *lǐ* (礼) (Thomas‐Walters, Cheung, et al., 2020). These values intrinsically affect behavioral interactions in Chinese society and are intertwined with social concepts that guide relationships and network connections, *guānxì* (关系), and the maintenance of face, *miànzǐ* (面子). In Thailand, different values for ivory emerge. For example, in some Buddhist communities, an ivory pendant is believed to be auspicious and to provide spiritual protection (USAID‐WA, 2017, 2021). For elephant owners in Surin province, Thailand, broken pieces of ivory tusks are passed down through generations and are associated with sentiment and spiritual beliefs regarding protection from harm (Chaitae et al., 2023). Therefore, understanding the psychological and sociocultural drivers of ivory consumption is essential for targeted demand‐management strategies.

Social marketing is increasingly used to improve global conservation outcomes (Green et al., 2019) because it offers behavioral theory, strategies, models, and frameworks that can be applied in the development of robust conservation behavior change interventions (Veríssimo, 2019). Social marketing is based on decades of application and learning in the public health and international development fields (Lee & Kotler, 2011; Truong & Dang, 2017). Best practice social marketing principles are grounded in the benchmark criteria of the United Kingdom's National Social Marketing Centre (NSMC) (NSMC, 2007), which are adapted to examine the quality of application of the social marketing indicator (SMI) tool (Wettstein & Suggs, 2016). When applied, the principles associated with these frameworks serve as optimal conditions that incite behavior change. Social marketing clarifies that to determine the causal effects of specific interventions on consumer behavior, interventions must use rigorous observational, experimental, or quasi‐experimental designs (Byerly et al., 2018; Dunn et al., 2021; Duthie et al., 2017; Frondel & Schmidt, 2005; Moorhouse et al., 2022) and adequate counterfactuals (Ferraro, 2009; Ferraro & Pattanayak, 2006) for robust impact evaluation (Veríssimo et al., 2018). Currently, most evaluations are of self‐reported behaviors and performance metrics, which cannot demonstrate actual behavior change and provide only insight into awareness raising among target audiences (Sheeran, 2002).

We sought to understand how social marketing principles have been integrated in ivory demand management in China and Southeast Asia. This work was inspired by Greenfield and Veríssimo (2018), who found conservation organizations’ use of the social marketing principles was limited for ivory and rhino horn in China and Vietnam from 2005 to 2015. We collected diverse qualitative data in a literature review and conducted interviews with practitioners, including managers and consultants, of ivory demand interventions to determine what a robust and inclusive social marketing evaluation entails. We used a novel analytical framework in which we combined the NSMC benchmark criteria and applied the SMI. We provide examples of recent interventions to explore the use of social marketing principles for future implementors to learn from. We used the literature review and interview results to address whether consumer‐focused demand‐management interventions had the potential to affect ivory consumer behavior and to examine how key social marketing principles are operationalized in recent ivory consumer behavior interventions.

## METHODS

### Literature review

We conducted a broad qualitative review of primarily gray literature written in English and Chinese to collect data on consumer‐focused ivory demand‐management interventions in China and Southeast Asia. Moving away from the term *demand‐reduction campaign*, we refer here to the behavior change efforts that influence consumers as *demand‐management interventions*. This term accounts for interventions aiming to not only reduce demand but also affect demand through stabilization or promotion of sustainable alternatives (Hinsley et al., 2022; Veríssimo, ‘t Sas‐Rolfes, et al., 2020; Veríssimo, Vieira, et al., 2020). Adapted from Veríssimo and Wan (2019), we define an ivory demand‐management intervention as the outreach activities implemented to incite voluntary change in the current or potential behaviors of ivory consumers. Our inclusion criteria required an intervention to have a known start year, an identified management organization or organizations, and a country or regional demographic associated with its target audience.

We established an initial list of all potential interventions from Sharif (2014), the United States Agency for International Development's (USAID) Reducing Demand for Wildlife program (USAID‐RDW, 2017), and Veríssimo and Wan (2019), which resulted in 21 relevant interventions released from 2008 to 2015. To update this review, we searched the TRAFFIC‐managed Change Wildlife Consumer resource library (www.changewildlifeconsumers.org/resources) and retrieved all available records (*n* = 146) in October 2022. Where available, we downloaded each record and searched for the following English keywords: *ivory*, *elephant*, and *demand*. In Chinese language documents, we searched for equivalent keywords: *象牙*, *大象*, and *需求*. This resulted in 107 searchable documents after removal of duplicates and records containing missing or broken URL links. Upon the identification of an intervention, the name, tagline, slogan, or key imagery was searched in Google (English text), Google Scholar (English text), Google Lens (reverse search by image), and Baidu (Chinese text). We reviewed up to the first 20 results displayed in the search engines. We found relevant information in gray literature sources mostly composed of annual reports, campaign reports, organizational digests, blogs, news articles, films, online games, apps, social media, surveys, case study reports, and evaluation reports. We finalized a list of 75 interventions for analyses (Appendix S1).

### Interviews

The available evidence was largely from the gray literature; thus, to fulfil our first research objective, we conducted supplementary expert semistructured interviews to gather supporting and contextual information from practitioners involved in the design, implementation, monitoring, and evaluation of ivory demand‐management interventions. Given the extensive period the interventions covered, we prioritized conducting interviews with organizations active in the most recent 5‐year period, when staff turnover was likely to have less impact on the knowledge of the design and implementation of the interventions. We found 23 organizations had leading roles in intervention roll out across the 15 years. Four organizations—WildAid, the International Fund for Animal Welfare, USAID Wildlife Activity (USAID‐WA), and the different country branches of the World Wide Fund for Nature (WWF)—contributed to at least 48 interventions originally identified. Therefore, we prioritized interviews with these organizations.

We used a purposive sampling strategy to contact 9 organizations, which resulted in 4 participants, 2 invitation declines, and 3 unresponsive contact requests. Organizations were contacted by email to participate in an interview. First, they were asked to complete an online screening survey to ensure they were appropriate to participate and to identify which of the 75 interventions they had sufficient knowledge to be able to discuss. An opportunity to provide details on interventions not already listed was also included in the survey. In the screening survey and during interviews, snowball sampling was used to find other appropriate practitioners, which led to one further interview. Our interview participants were primarily senior practitioners whose collective experience covered our full intervention time span from 2008 to 2022 (Table 1). In our results, interviewees are coded as P1 to P5 (Table 1). This project was approved by the Biology Ethics Committee at the University of York (reference code CB202301). All ethical requirements were followed, including receiving free, prior, and informed consent from all interview participants. Interview questions and the privacy notice are in Appendix S3.

**TABLE 1:** Details of interviews with ivory demand‐management practitioners.

Interviewee^a^	Length (min)	Organization type	Organization size	Role	Country expertise
P1	100	Nongovernmental organization or charity	Large (>250 employees)	Regional director	China
P2	69	Government agency	Large (>250 employees)	Consultant	Thailand, China, and Vietnam
P3	85	Nongovernmental organization or charity	Small (<50 employees)	Program director	Thailand
P4	76	Nongovernmental organization or charity	Small (<50 employees)	Program director	China
P5	56	Multiple relevant roles in NGOs and government agencies	Multiple (small and large)	Freelance consultant	Thailand and Vietnam

^a^ Respondents P3 and P4 were from different country branches of the same organization. They were not informed of either party's involvement.

### Social marketing data analyses

**FIGURE 1:**
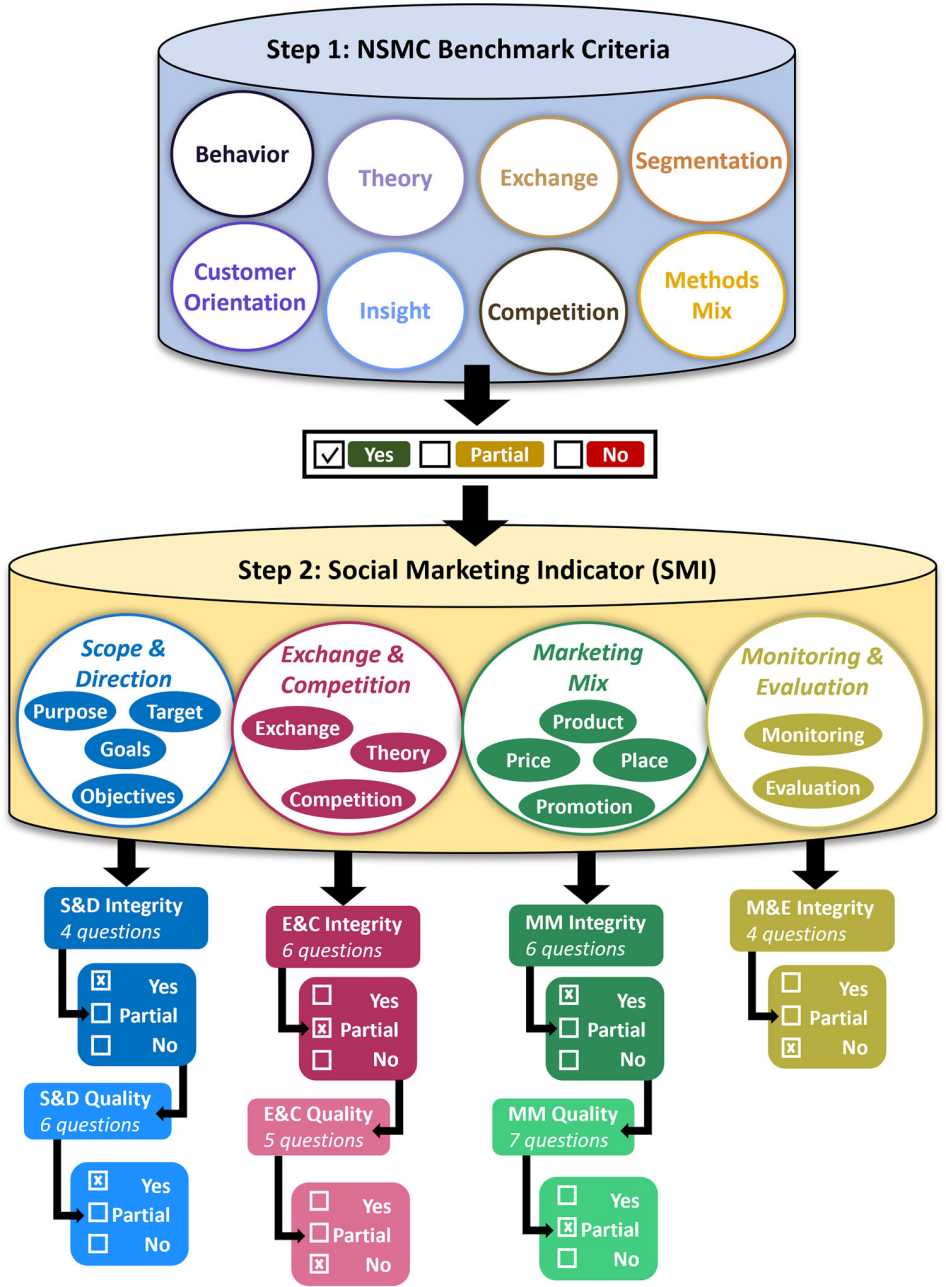
Analytical framework used for the social marketing assessment of consumer‐focused ivory demand‐management interventions. The adapted social marketing indicator (SMI) was originally by Wettstein and Suggs (2016) and has 38 questions pertaining to the 4 components shown.

We developed a qualitative analytical framework based on key principles of social marketing to assess the interventions (Figure 1). The framework consisted of 2 parts: first, the widely used 8‐point NSMC benchmark criteria (hereafter, benchmarks), based on Andreasen's original 6 benchmarks (Andreasen, 2002; NSMC, 2007), and, second, the SMI tool by Wettstein and Suggs (2016).

The benchmarks are guideline prerequisites for successful behavior change interventions and include the criterion of behavior, customer orientation, theory, insight, exchange, competition, segmentation, and methods mix. To qualitatively evaluate the interventions with the benchmarks, we created a reference guide based on established definitions and guidance of each benchmark (available from www.thensmc.com/content/nsmc‐benchmark‐criteria). Interventions contained different communication components and delivery mechanisms, such as pledges, which were analyzed as a form of augmented product—meaning they created supplementary benefits to enhance the core product (i.e., the campaign message) and distinguished it from similar products offered by the competition (Costello et al., 2022) (Appendix S2). The collated information for each intervention was then assessed against this reference guide to identify evidence of integration of each benchmark. Each intervention was scored on a discrete scale of 0–2 to reflect how well the benchmarks were integrated. A score of 0 reflected either no integration of the benchmark in the intervention or that no data were available for assessment. A score of 1 was awarded when some or partial evidence was available and it seemed more likely than not that the principles of the benchmark had been considered based on the available evidence. A score of 2 was awarded when there was clear evidence that there had been purposeful consideration of the benchmark.

The SMI tool provides critical insight into the application of social marketing principles via 38 questions centered around 4 clusters. Each question is associated with 1 of 13 steps defined as best practice principles in social marketing (details on each cluster and step are in Appendix S2). The clusters and associated steps are the scope and direction cluster (steps: purpose, target, goals, and objectives); exchange and competition cluster (steps: theory, exchange, and competition); marketing mix cluster (steps: product, price, place, and promotion); and monitoring and evaluation cluster (steps: monitoring and evaluation). The first 3 clusters are assessed along 2 lines of enquiry: process integrity, focused on adherence, and process quality, focused on the evidence basis. The SMI tool is limited by the monitoring and evaluation cluster where only process integrity is assessed (i.e., only confirms whether processes existed, provides no insight into quality). In the original SMI, there is a binary yes or no answering system for each question. However, due to data scarcity in this field (Greenfield & Veríssimo, 2018), we added a third middle‐ground partial answer to indicate that the evidence was weak and may not be well‐integrated in the intervention design despite some evidence such that the question could be answered in the affirmative.

For each of the SMI steps, we added a supplementary analysis conducted in R, which assessed the significance of temporal changes on effective inclusion with a generalized linear model with quasi‐binomial link (R Core Team, 2024). We compared interventions implemented before and after China's domestic ivory ban that started at the end of 2017. We treated full integration as 1 and partial integration as a numeric score of 0.5 and fitted separate models for each step and a final model with the average integration score across all 13 SMI steps (all raw data are available from the GitHub repository at https://github.com/MollyBrown96/social_marketing_ivory).

## RESULTS

Supporting contextual information from interviews (with individuals we identified as P1 to P5) is embedded throughout the results. Additional examples are in Appendix S4.

### Integration of social marketing principles

Over the 15‐year period (2008–2022), more interventions were implemented in China (Mainland and Hong Kong SAR) (*n* = 29) than in Thailand (*n* = 13), Vietnam (*n* = 5), Japan (*n* = 2), or Lao PDR (*n* = 1). Five more interventions were implemented on a global scale (with Asia‐specific communications) via digital social media channels (*n* = 2), a multi‐country exhibition including China (*n* = 1), and online documentaries (with Chinese dubbed versions) (*n* = 2). Most interventions were implemented from 2013 onward (*n* = 53). In 2008 and 2009, there was one intervention in each year.

### Awareness raising and behavior change

From 2008 to 2017, 65% (*n* = 19) of interventions did not integrate 4 or more benchmarks (Appendix S5) (e.g., Green Passports [2009], 96 Elephants [2013], NBA Cares [2013], We Must Act Now [2014], Elephant New Year: Join the Herd [2016], and Ivory Free Vietnam [2017]). We found basic segmentation of audiences, with blanket approaches used to influence general population demographics. Most notably, we found no integration of theory in 28 of 29 interventions implemented from 2008 to 2017.

Most interventions simultaneously attempted to educate, raise awareness, and advocate for policy change to influence consumer behavior. Respondent P2 (international consultant, government agencies) corroborated this finding: “most of the campaigns to reduce demand [at this time] were actually general awareness‐raising campaigns, which only focused on the general public.”

From our SMI analyses, prior to 2018, we found a dearth of principles integrated across many SMI steps (Appendix S6). We found a cascading effect triggered seemingly from a lack of research and segmentation of consumer groups affecting the SMI steps of target, objectives, exchange, and competition, which all require insight into the specific behaviors of a segmented audience for high‐quality integration. Therefore, due to the lack of behavioral research and segmentation, most steps were poorly integrated, diminishing the potential for behavior change.

### Developing adoption of strategic behavioral decision‐making

**FIGURE 2:**
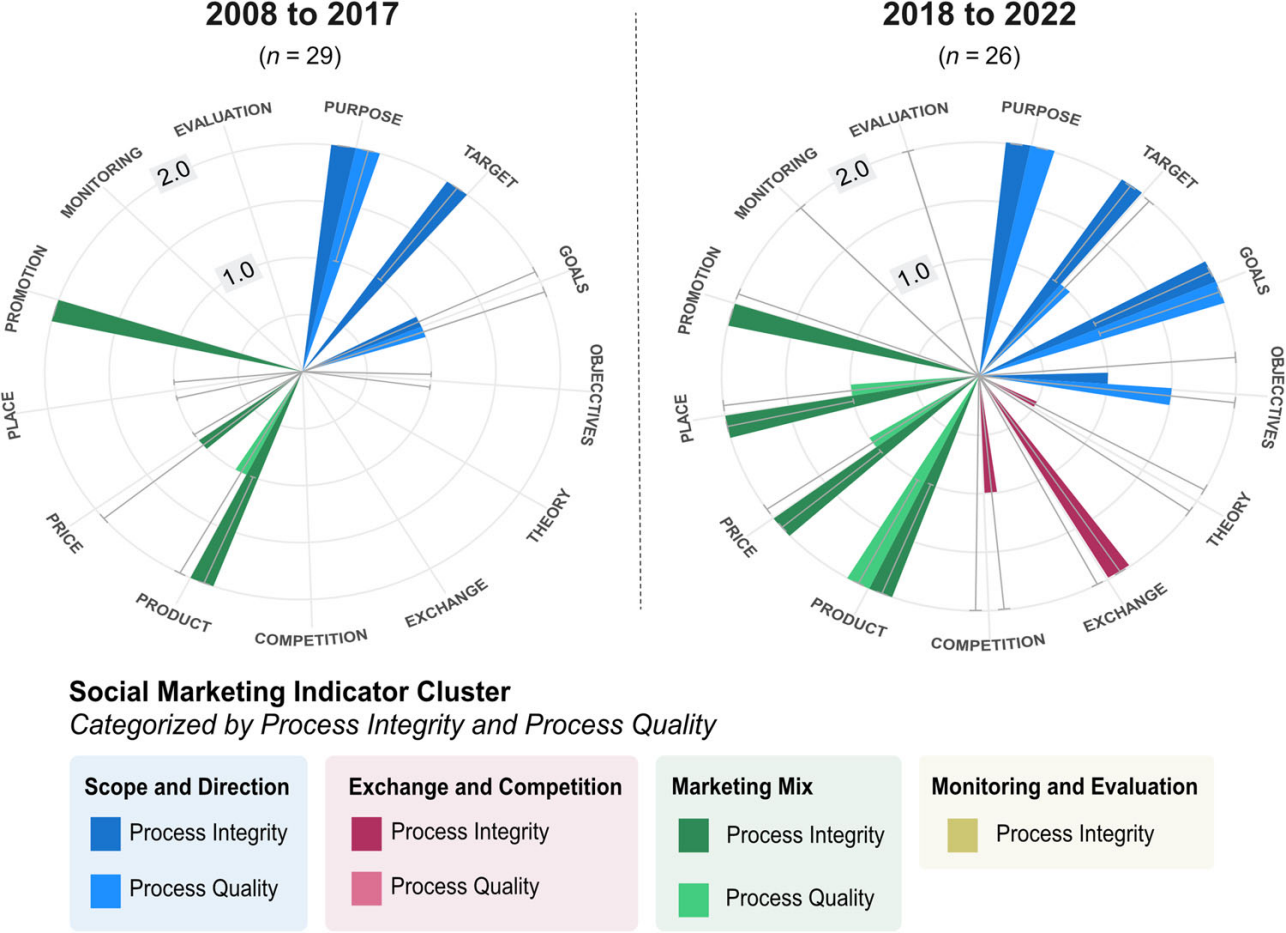
Average (median [see below]) scores from a social marketing indicator (SMI) analysis of ivory demand‐management interventions (*n* = 55) in China and Southeast Asia from 2008 to 2022: (a) 2008–2017 (*n* = 29) and (b) 2018–2022 (*n* = 26). The SMI consists of 4 clusters (scope and direction, exchange and competition, marketing mix, and monitoring and evaluation) made up of 13 steps (components along the outside of the circles) (scope and direction cluster steps: purpose, target, goals, and objectives; exchange and competition cluster steps: theory, exchange, and competition; marketing mix cluster steps: product, price, place, and promotion; monitoring and evaluation cluster steps: monitoring and evaluation). The first 3 clusters have a 2‐tone color split that indicates the total average scores for each step's process integrity (darker color) and process quality (lighter color), whereas monitoring and evaluation only includes process integrity. The SMI median scores are on a discrete range from 0 to 2, with the scale points 1.0 and 2.0 depicted (error bars, interquartile range [0.25 and 0.75]).

A key finding of this study was the increased integration of social marketing principles over time (Figure 2). We found a significant increase in average score of interventions from 2008 to 2022 (average logit increase in full inclusion 0.166 [SE 0.046], *χ* ^2^ = 3.652, df = 1, *p* < 0.001). From 2018 to 2022 specifically, we found a broad uptake of social marketing principles across the 26 interventions (Figure 2). Interventions from 2018 to 2022 had higher average scores (Figure 2b), and the principles of theory, monitoring, and evaluation were poorly integrated over time. Ten interventions (38%) integrated all benchmarks to some extent (i.e., gaining a partial or full score). These interventions targeted more specific behaviors, beliefs, and values (e.g., in Buy 1, Get 15 [2020], gifting behaviors were targeted; in Spiritual Beliefs [2019], messaging focused on beliefs of spiritual protection from harm; and Our Shared World [2022] focused on community values for collective harmony). Despite this increase of social marketing principles generally, approximately 26% (*n* = 7) of interventions were still devoid of 4 or more benchmarks. Theory remained the least integrated; only 13 interventions (50%) used some type of theoretical approach. However, these were often vague references to theory rather than detailed application. Benchmarks of segmentation, competition, and exchange were all equally integrated to some extent in 18 interventions (69%), whereas customer orientation (81%, *n* = 21), behavior (89%, *n* = 23), and methods mix (92%, *n* = 24) were integrated the most frequently.

We found the increase in the adoption of social marketing principles reflected in the experiences of interviewees who perceived a shift from experiential learning by doing to adopting more evidence‐based approaches (P1, P2, P5). This shift enabled intervention design to target more specific groups and behaviors; however, it did not take place due to adopting a social marketing approach. Respondent P2 highlighted the adoption of social and behavior change communications (SBCC) theory “to focus on specific aspects of the planning process” for demand management. Respondent P1 emphasized that over time there have been “three interconnected pathways to behavior change” that form the “behavior change framework” for ivory demand management in China. These pathways involved raising awareness, political advocacy, and social mobilization, the latter of which was emphasized as the ongoing phase across interviewees (P1, P3, P4, P5).

The USAID Wildlife Asia and Reducing Demand for Wildlife (USAID‐WA/RDW) SBCC approach (USAID‐RDW, 2020), adapted from the Communication for Change project (McKee et al., 2014), enabled strategic decisions based on consumer evidence insights. For example, interventions targeted more specific audiences and behaviors, such as the buying of ivory jewelry by wealthy Thai women in the 2019 Beautiful Without Ivory intervention and the buying of ivory trinkets from curio markets abroad by Chinese tourists in the 2022 Wildlife Free Traveler intervention. However, beyond grounding the use of consumer insight‐driven approaches, USAID‐WA/RDW lacked theoretical underpinning, or reporting of such, at the intervention level.

### Operationalization of social marketing principles in recent interventions

We found an increased integration of social marketing principles (process integrity) and an improved evidence base guiding the design and implementation of interventions (process quality) in interventions since 2018 (Figure 2).

### Scope and direction principles (SMI Cluster 1)

Consumer groups were targeted with more specificity than in previous years due to primary and secondary consumer research providing behavioral insight into different group's motivations and barriers for ivory consumption. This approach increased the integration of all steps in the SMI's scope and direction cluster. For example, in Say No to Shipping Illegal Wildlife Products (2022), the behavior of courier delivery drivers who accepted packages suspected or known to contain IWT products was targeted (WildAid, 2022). This tapped into compelling social factors that could result in loss of job security and moral integrity, which increased the likelihood of a behavioral effect from the intervention's targeting.

In Digital Deterrence (2018), digital precision marketing was used to target 3 different online ivory consumer groups, each with strategic communication materials and delivery mechanisms (Appendix S2). For example, the group identified as the most likely ivory consumers were shown strong warning messages that suggested surveillance of their online searches and highlighted the punitive ramifications of illegal ivory purchasing (i.e., to “destroy the sense of online anonymity… and increase the perception of personal risk” [P2, consultant, multiple countries]). In Beautiful Without Ivory (2019) and its follow‐up intervention Elephants Wear Ivory Best (2022) (Appendix S1), consumer groups were segmented via communication strategies based on formative research. This research found well‐educated, affluent urban Thai women were key purchasers of ivory for its aesthetic (personal adornment) and spiritual value (harm prevention). The target group was identified using segmentation of consumers who already own ivory, those who desire to own ivory accessories, and those who believe it brings good luck.

### Exchange and competition principles (SMI Cluster 2)

**FIGURE 3:**
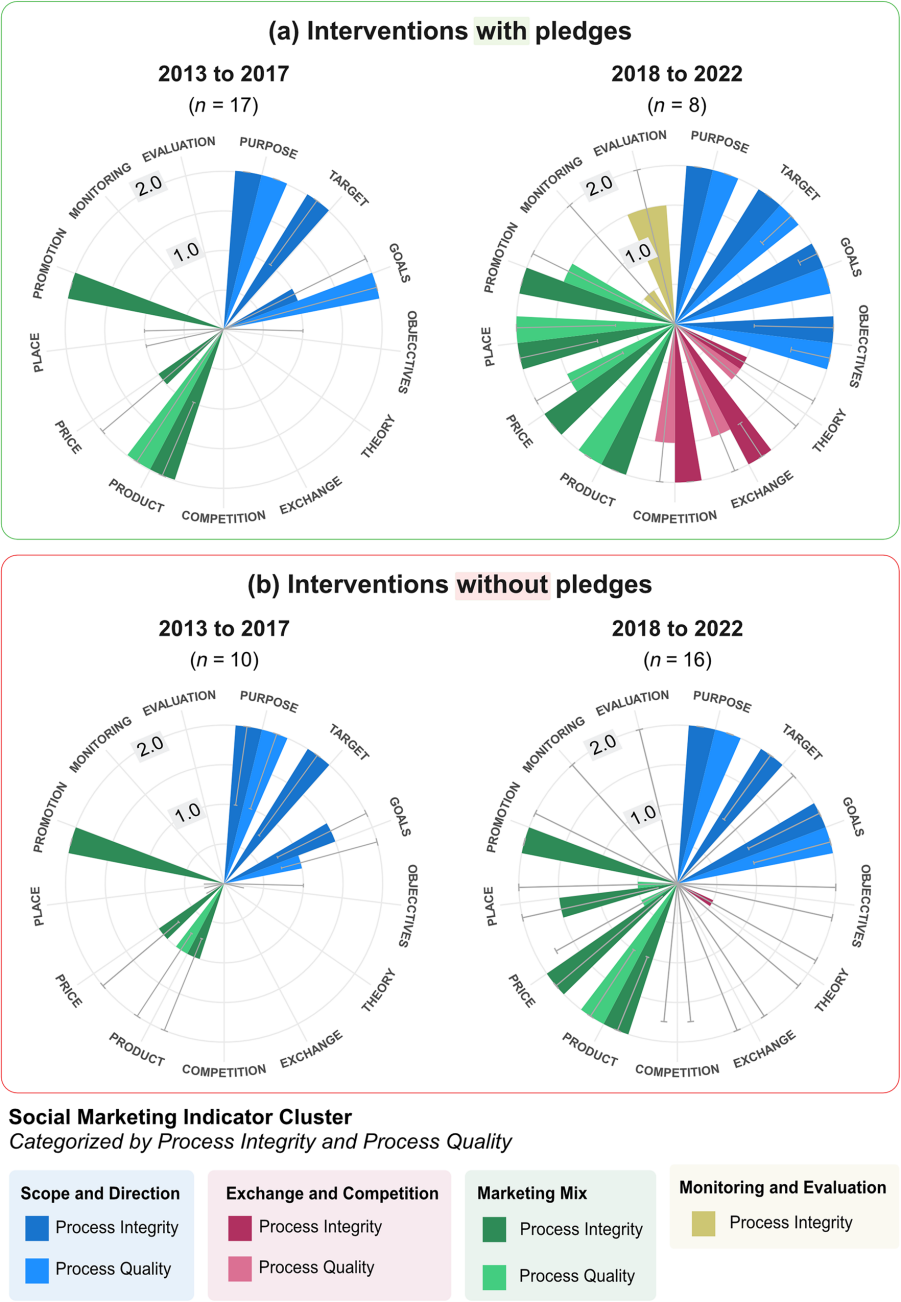
Average (median [see below]) scores from the social marketing indicator (SMI) analyses for ivory demand‐management interventions implemented from 2013 to 2022 (a) with a pledge to change their behavior and (b) without a pledge (definitions of cluster and steps provided in Figure 2’s legend). The SMI median scores are on a discrete range from 0 to 2, with the scale points 1.0 and 2.0 depicted (error bars, interquartile range [0.25 and 0.75]).

Another of our key findings was the importance of exchange and competition principles for wider integration of social marketing principles. We found this specifically through the inclusion of a pledge element, which corresponded to greater overall integration of social marketing principles (Figure 3). For example, in Mercy is Power (2021), the public pledged to not buy ivory amulets after being asked to choose an amulet style to use to worship from an online list. They were also asked to choose the most inspiring example from a list of auspicious phrases. These choices personalized an e‐gift of a digital *yantra* (a powerful mystical diagram) received in exchange for their commitment. However, because we only found a correlation between pledges and increased integration of exchange and competition principles, we could not infer causation. Thus, it is plausible that pledges forced the design stage of an intervention to recognize the different behaviors that motivate and inhibit the target behavior of the pledge.

We found minimal evidence of how behavioral theory influenced intervention strategies. For example, the diffusion of innovation theory that explains how ideas spread through social systems (Rogers, 2003) was reported in USAID‐WA intervention design but was not supported by explanation of how it influenced specific decision‐making. This was also reflected in the SBCC approach, which relied broadly on a socioecological model. We recorded no integration of a step or principle, when there was no available evidence. However, this does not necessarily mean that consideration for, or integration of, the principle did not happen; it was just not clear from our sources.

Applying theoretical approaches was perceived broadly by practitioners as not practical due to the rapid timeline expectation for project delivery. Respondent P3 explained that interventions have “next to no time for design, as we need to start implementation.” However, based on collaborative opportunities such as TRAFFIC‐led workshops (TRAFFIC, 2016), some practitioners thought they had gained theoretical understanding, which helped focus interventions on specific consumer behaviors. For example, P3 noted learning from such experiences that “we have to change our message and try to tackle the motivations,” which demonstrates a potentially increasing capacity in the demand‐management community to use social marketing principles. However, overall, we found evidence indicating a significant dearth of applied social marketing theory in ivory demand management.

### Marketing mix principles (SMI Cluster 3)

The steps of the marketing mix were, as expected, integrated more frequently than other SMI elements. However, the steps were poorly applied (i.e., research underpinning the choices made was not robust) (Appendix S2). The primary reason for the poor process quality of the marketing mix was due to a lack of pretesting of most interventions. The Spiritual Beliefs (2022) project, however, did apply pretesting and adapted messaging materials, from direct to indirect, to better appeal to the target audience.

### Monitoring and evaluation principles (SMI Cluster 4)

Since 2018, only 9 interventions integrated both monitoring and evaluation to some extent (Appendix S2). Behavioral process and impact evaluation evidence was widely anecdotal and consisted of self‐reported behavior change, such as in Wildlife Free Traveler (2022) and Wildlife Free Gifting (2020). Two interventions, Contraband Customs Campaign (2019) and Mercy is Power (2021), conducted postimplementation evaluations. However, we found no evidence that indicated monitoring or adaptation had taken place during the intervention; therefore, no change in consumer behavior could be assessed. We found no evidence of an adequate counterfactual used as part of the design process for any intervention or causal impact evaluation approaches. From interviews, however, monitoring and evaluation practices were reported as approaches used to determine the reception of interventions by target audiences, and it was reported that this information was used to inform more strategic decision‐making relative to target groups and their specific behaviors.

## DISCUSSION

### Leading with behavioral science and evidence

Before 2018, interventions were unlikely to affect the behavior of ivory consumers due to a lack of specific behavioral focus, too broad target audiences, and the dominant use of one‐size‐fits‐all blanket messaging (Thomas‐Walters et al., 2021). Making the general public the target of an intervention (e.g., the Say No to Ivory and Rhino Horn [2013]) contradicts foundational principles of insight‐driven segmentation for behavior change (Kubacki et al., 2017). We found a disconnect between social marketing and intervention design from 2008 to 2017 that severely limited the likelihood of ivory consumer behavior change in earlier years (Aceves‐Martins et al., 2016; Carins & Rundle‐Thiele, 2014). This finding is supported by Greenfield and Veríssimo (2018), who found theory‐based approaches are seldom adopted.

More recent ivory interventions based on insight‐driven approaches, in which consumer motivations are researched and addressed through strategic design and implementation decisions, provide greater chance of behavior change (Doughty et al., 2020). However, due to limited use of behavioral theory, counterfactuals, and causal inference methods, there is no clear evidence of an intervention directly changing ivory consumer behavior. The lack of theoretical evidence may be skewed by the poor reporting standards of most interventions and is a wider limitation across conservation marketing field (Veríssimo & Wan, 2019). This limited our understanding of how specific behavioral theories were used or adapted for deeper behavioral insights within the interventions (USAID‐RDW, 2022a). Despite a correlation existing between the uptake of social marketing principles and the use of SBCC approaches over time, we found a clear gap in understanding of the causative nature of what elements of SBCC theory and practice may lead to behavioral change. This is the critical challenge with the current state of ivory interventions and must be addressed in future interventions.

We see prime examples of robust behavioral research conducted for other IWT products in China and Southeast Asia to draw from. One behavioral‐informed approach conducted by Zheng et al. (2024) sought to diminish demand for endangered turtles kept illegally as pets in China by applying a mixed‐methods approach that identified the capability, opportunity, and motivations of behavior (i.e., the COM‐B model [Michie et al., 2011]). Underpinned by COM‐B, the frameworks TESTS (target, explore, solution, test, scale) and EAST (easy, active, simple, timely) (BIT, 2014, 2022; Rare & BIT, 2019) were used to build consumer profiles to determine target audiences, possible entry points for intervention delivery and format, and thematic analysis of key drivers and perceptions of trade and consumption conditions (Zheng et al., 2024). We found multiple missed opportunities to rigorously test, adapt, and reform ivory demand management for effective behavior change through high standards of behavioral science found in social marketing for effective contribution to global elephant conservation strategies.

### Behavior change capacity of ivory demand‐management interventions

Our results showed a clear subset of 2 SMI clusters and multiple benchmarks, which were frequently missing from the design and implementation processes. These principles include theory, exchange, competition, monitoring, and evaluation. The importance of these concepts to actualizing behavior change in consumers cannot be underestimated (Akbar et al., 2022). Each of these principles requires different considerations to achieve meaningful integration.

Interpretation and application of behavioral theories are required to link relevant theoretical determinants to intervention choices, moving beyond lip service to use of behavioral theory as identified, which we saw in recent interventions for ivory (Hagger & Weed, 2019). Theory can also help bridge the gap for practitioners more familiar with traditional marketing communications (the field of many demand‐management practitioners) by creating a common language through theory providing “a coherent and explicit framework for designing, evaluating, and optimizing interventions” to influence sustainable behavior changes (Dalgetty et al., 2019, p. 352). For example, when behavior is genuinely considered for ivory consumers, the psychological, social, cultural, and physical drivers and barriers that lead to consumption can be considered, allowing other benchmarks to be considered thoroughly.

Based on the finding that pledges can increase the integration of social marketing principles, it is worth practitioners taking a broader and more creative view on intervention components. Augmented products, such as pledges, petitions, social media initiatives, gifting guides, and mobile apps, could help reinforce other social marketing principles by issuing a specific need for the interventionist to consider the costs and benefits of the behavior (i.e., core product) for the target audience. Pledges usually incite a call to action of a specific behavior—a key social marketing method (Smith, 2006). Therefore, in the process of designing a pledge, audiences’ behaviors can be more specifically targeted, providing opportunity for social marketing principles of exchange and competition to be more deeply integrated. These products are repositioning focused, which can provide encouragement, remove barriers, or sustain the desired behavior (Lee & Kotler, 2011). For ivory, augmented products could, for example, be expert consultation from master carvers to collector audiences (Burton, 2019), a telephone helpline for recipients to declare receipt of (legal) ivory gifts (i.e., not when gifting is used to conceal illegal purchasing activity), which is currently not monitored or regulated based on China's ivory ban that precludes gifting or inheritance practices from punitive action (Chen et al., 2023), and virtual 3D technology ivory workshops (i.e., similar to those run as part of a Guangzhou University course [Gao & Xu, 2022]) to help promote a new generation of Chinese craftsmanship in which sustainable alternatives are used.

Monitoring and evaluation were the least integrated social marketing principles throughout the 15 years of interventions. Social marketing literature implores the robust use of monitoring and evaluation procedures (Lee & Kotler, 2011). When these processes are not defined early on in the design phrase, the appropriate resources, budget, and time cannot be procured. Monitoring includes both pretesting prior to implementation (piloting) and then systematic monitoring from the start and during implementation. This is crucial for IWT market‐side interventions; piloting provides necessary insight for adaptation (Ferraro & Pattanayak, 2006; McKenzie‐Mohr & Schultz, 2014; Zhou et al., 2021). Foremost, monitoring provides the data essential for robust evaluation and future learning opportunities to adapt and improve throughout the life cycle of an intervention (Doughty et al., 2021; Hinsley et al., 2022; Venturini, 2016).

### Diversifying intervention strategies and delivery mechanisms

The poor quality of intervention data and the dominance of social mobilization and SBCC approaches in ivory demand management limit the potential for long‐term behavioral change. Increasing attention has been paid to adopting behavioral science approaches to tackle IWT (Veríssimo et al., 2024), including use of robust evaluation methods for other IWT products (Baylis et al., 2016; Doughty et al., 2021, 2020; Thomas‐Walters, Veríssimo, et al., 2020). However, for ivory, due to a lack of diverse strategic and theoretical approaches, we found a rinse‐and‐repeat nature in the communications‐focused interventions implemented. We established that the basic principles of behavior change from a social marketing perspective have developed over time. However, the quality of integration and persistent lack of behavioral theory, monitoring, and evaluation raise concerns for the likely effectiveness of interventions to change persistent behaviors that lead to ivory consumption, such as for gifting and personal collecting.

In social marketing, only social mobilization from the behavior change framework promoted by interviewees could be conceived to be attempting behavior change. It is seen as one supportive measure used when the framing of an intervention is more flexible and inclusive and thereby able to attract and potentially mobilize a larger group (Daellenbach & Parkinson, 2017). However, in this instance, conceptually, social mobilization more closely follows McKee's (1992) broad definition, which encompasses all potential stakeholder groups (e.g., government bodies, corporations, social and religious leaders, and the target audience themselves) as targets for mobilization via any delivery mechanism (e.g., mass media, lobbying, and training). This definition developed from social change programs in developing countries, where intersectoral alliances and government support may have been weak for behavior change interventions (Donovan & Henley, 2010). Therefore, for ivory demand management in China and Southeast Asia, the conceptual reliance on delivering effective demand management at scale through social mobilization does not follow the targeted, insight‐driven approaches typically described in social marketing.

Interventions should aim to affect a more diverse range of consumption behaviors that contribute to overall ivory demand (Gao & Clark, 2014). Currently, nonpurchasing behaviors involved with ivory consumption, such as gifting, ownership, storage, and disposal, are seldom targeted. Narrowly focusing only on purchase and sale is likely to have limited effect on the other behaviors that sustain demand, particularly given the importance of ivory's symbolic value as a form of social capital and varying values of ivory for different consumer groups (Meijer et al., 2021). For example, gift‐giving behavior encompasses the acquisition, exchange, and use of gifts between participants and establishes a reciprocal cycle (Clarke, 2007). The comprehensive gift‐giving research review by Givi et al. (2022) discusses cultural differences, gifting occasions, giver–recipient relationships, individual‐level differences, and the interpersonal nature of the process as critical factors affecting gift giving. For intervention strategies to affect deeply symbolic social values, we recommend considering how each of these behavioral phenomena is embedded in ivory gift giving. These theories may help guide intervention design beyond considering only the responsibility of the individual consumer to considering the powerful, socially constructed values related to ivory. The legal trade in mammoth ivory in China may provide a route to investigating ivory's symbolic value for consumers with applications for elephant ivory products (Farah & Boyce, 2019).

In China particularly, the widespread awareness‐raising initiatives of the past decade should empower the demand‐management community to target highly segmented consumer audiences with specific interventions. Systems‐thinking approaches increasingly used in social marketing literature and practice could support this shift (Domegan et al., 2017; Gallegos et al., 2023; Mahajan et al., 2019). Systems thinking is described as a way of thinking and understanding that considers the elements, interconnections, and function or goal of things (Mahajan et al., 2019; Parkinson et al., 2025). Adopting a systems‐thinking approach for intervention design could therefore help pinpoint which interactions in the complex systems of ivory behaviors could be targeted more acutely within the wider operating landscape of law enforcement and environmental education interventions for ivory consumer behavior change impacts.

### Study limitations

Poor reporting standards limited the data on ivory demand‐management interventions. This increased the subjectivity of our analyses. Despite using mixed methods to increase the robustness of data collected, we could not find evidence for or against the integration of every social marketing principle. When evidence was not available that the principle was not integrated, it could have been the result of poor reporting: an important lesson for behavioral change practitioners to learn. Further, the management organizations’ lack of transparency in the design and implementation of interventions caused challenges by reducing the data available for analysis. Accessing the appropriate intervention practitioners to interview was often a challenge. Changes in the organization's staff and program structures meant in some instances current staff were not familiar with the intervention in question and institutional memory had been lost. Some interviewees deferred their own expertise to that of their senior management or another organization entirely. This was a limiting factor because we sought the experience of a range of practitioners involved in the intervention design and implementation and primarily interviewed more senior members. Classifying evidence with the benchmarks and the SMI frameworks required some subjective judgment, but the parallel use of these frameworks generated information about their respective strengths and weaknesses and substantiated our understanding of the quality of the interventions. When using the SMI, careful attention needs to be paid to the weighting of different elements in interpretation of the results because this is not accounted for in the tool itself. Each answer was weighed equally, so dissecting which principles were most important for different interventions requires familiarity with the social marketing literature (Wettstein & Suggs, 2016).

### Future directions

Demand‐management interventions for ivory should strive to build on the growing momentum for practitioners, academics, and management organizations to collaborate on the design and implementation of consumer interventions. Knowledge‐exchange networks, such as the Community of Practice established in 2016 by TRAFFIC (TRAFFIC, 2018) and the Thailand Demand Reduction Steering Group, founded in 2020 and expanded in 2022 by a network of practitioners (USAID‐RDW, 2022b), were commented on in interviews as useful touchpoints for practitioners to coordinate campaigns and exchange expertise. Therefore, greater financial and logistical support for knowledge exchange and collaborative initiatives needs to be established to enable wider participation in behavioral science‐led interventions. The social marketing community's formal associations (e.g., the International Social Marketing Association, https://isocialmarketing.org) and its regional branches should be more widely engaged with to bridge academic–practitioner challenges. Associations provide strong domestic and international networks for knowledge exchange, training, and collaborative opportunities relevant for IWT demand‐management interventions, particularly for bridging theory–practice application gaps, and cost‐effective monitoring and evaluation approaches. Greater participation in the field of social marketing by nongovernmental organizations (NGOs), government bodies, and other implementation organizations would help broaden and level the behavioral science knowledge of intervention stakeholders and prevent only senior practitioners or those with public health expertise from being familiar with behavioral theory, as we found in this study.

Participatory methods and codesign principles remain underused in IWT consumer research and demand‐side interventions (Hübschle & Margulies, 2024). Lefebvre (2012) argues that codesign is an essential part of any social marketing intervention and should be used to empower citizens through the collaborative processes that allow people to design programs for themselves and people like them (Schmidtke et al., 2020). Codesign methods may provide new insights into the challenging context of researching illicit or sensitive behaviors (Hu et al., 2023). For example, to understand what sparks enthusiasm for ivory tourism or inhibits those who are susceptible to spontaneous ivory consumption, codesign approaches could be used to segment traveler‐focused interventions more specifically based on inductive insights from consumers. It is important to explicitly recognize the dynamic interrelationships that exist within social systems to engage different stakeholders in codesign processes (Lefebvre, 2012). For example, the often communication‐focused strategies of NGOs could be strengthened by coordinating codesign methods with a wider array of stakeholder groups (Dietrich et al., 2016). Working with funders and implementation partners, including government bodies and media agencies, could inform creative and evidence‐based approaches to inform codesigned behavior change interventions (Kerr et al., 2022).

Research directions need to continue to address the causal impact of interventions and build understanding across behaviors in micro (i.e., individuals and one‐on‐one interactions), meso (i.e., organizations, groups, and communities), and macro (i.e., governments, institutions, and societies) levels of society. This work should seek to map the direct and indirect impacts of behavior change interventions on ivory consumption for a more robust understanding of drivers and barriers to demand (Jones & Shreedhar, 2024). This would feed back into developing more consumer‐driven insights required for demand‐management interventions to be effectively designed according to social marketing principles. For example, the research conducted on Japan's declining ivory demand by Thomas‐Walters et al. (2023) established a clear causal understanding of the social and economic processes that lead to behavioral changes toward ivory consumption in Japan. In contrast, Mahajan et al. (2019) used a systems‐thinking approach to evaluate the causal drivers of ivory demand in China and the impacts of interventions. Therefore, incorporating causal evaluation approaches in the design of an intervention could help unravel the complex variables affecting ivory demand.

## Supporting information



Supporting Information

Supporting Information
