# Supplementary material for: Assessing the integration of social marketing principles in ivory demand management interventions in China and Southeast Asia
Source: Conserv Biol. 2025 Dec 16;40(2):e70191. doi: 10.1111/cobi.70191 (PMC13036303; doi:10.1111/cobi.70191)
Supplement: Supplementary file 2 — Supporting Information [file COBI-40-e70191-s001.docx]

# APPENDIX: SUPPORTING INFORMATION

**S1. Interventions List**

| **Interventions included in analysis (ID 1 to 55)** | | | | | | | |
| --- | --- | --- | --- | --- | --- | --- | --- |
| **ID** | **Start Year** | **Intervention Reference**  **Name** | **Organization(s) involved** | **Country(s) involved** | **Target Audience(s)** | **Relevant URL** | **Initial Source(s)*** |
| 1 | 2008 | Mom, I Have Teeth Campaign | IFAW, JC Decaux Advertising | China | Chinese | <https://www.ifaw.org/projects/wildlife-crime-prevention-china/updates-from-the-field> | 2014, 2019 |
| 2 | 2009 | Green Passports Initiative | IFAW, Kenyan Embassy in China | China | Chinese | <https://www.yumpu.com/en/document/read/5339826/annual-report-1-july-2010-30-june-2011-international-fund-for-> | CWC/SE |
| 3 | 2013 | Schools United for Elephants Campaign | Eco-Sys Action Foundation | Hong Kong SAR | Hong Kongese | <https://vimeo.com/101940043?> | 2019 |
| 4 | 2013 | iThink Ivory Campaign | Freeland Foundation, Critical Ecosystem Partnership Fund, U.S. Agency for International Development, U.S. Fish and Wildlife Service | China, Vietnam | Chinese, Vietnamese | <https://www.cepf.net/sites/default/files/final-report-64071.pdf> | 2017, 2019 |
| 5 | 2013 | NBA cares campaign | WildAid, National Basketball Association | China | Chinese | <https://wildaid.org/nba-cares-and-wildaid-launch-campaign-in-china-to-save-africas-endangered-wildlife/> | CWC/SE |
| 6 | 2013 | Say No to Ivory and Rhino Horn Campaign | WildAid, Save the Elephants, African Wildlife Foundation, Yao Ming Foundation | China | Chinese | <https://wildaid.org/ivory-demand-reduction-campaign-launched-in-china/> | 2014, 2017, 2019 |
| 7 | 2013 | SMS text alert for all visitors to Kenya from China | Chinese Ministry of Foreign Affairs, China State Forestry Administration | China | Chinese | <http://ke.china-embassy.gov.cn/lsfw/zytz/201501/t20150130_7128412.htm> | 2019 |
| 8 | 2013 | 96 Ninety-six elephants Campaign | WCS, China Wildlife Conservation Association | Global | International | <https://www.wcs.org/96-elephants> | 2014, 2019 |
| 9 | 2013 | Vanishing Treasures campaign | TRAFFIC, WWF, Ogilvy & Mather | China | Chinese | <https://www.traffic.org/news/artist-makes-a-splash-with-vanishing-treasures/> | 2014, 2019 |
| 10 | 2013 | Wild and precious Exhibition | UNEP, CITES, GoodPlanet foundation | Global | International | <https://www.unep.org/news-and-stories/press-release/wild-and-precious-exhibit-travels-bangkok-nairobi-and-beijing> | 2014, 2019 |
| 11 | 2013 | Wildlife Crime: Don't be part of it! Campaign | UNODC | Global | International | <https://www.youtube.com/watch?v=I3jlt16LhPs> | CWC/SE |
| 12 | 2013 | End of the Wild Feature Film | WildAid | China | Chinese | <https://wildaid.org/resources/the-end-of-the-wild-trailer-feat-yao-ming/> | 2014, 2017 |
| 13 | 2014 | We must act now Campaign | UNEP, Save the Elephants, WildAid | China | Chinese | <https://www.robshumaker.com/good-works-elephants/> | 2014, 2019 |
| 14 | 2014 | AEFF film White Gold | AEFF | China | Chinese | <http://www.aemps.aeff.org/2014/01/01/white-gold-launched-in-the-united-states-and-china-to-help-stop-the-killing-of-elephants-in-africa/> | CWC/SE |
| 15 | 2014 | Bring No Ivory Home Campaign | WCS, Beijing Customs, WildAid, CITES Management Authority of China | China | Chinese | <https://newsroom.wcs.org/News-Releases/articleType/ArticleView/articleId/6781/Beijing-Customs-Launches-Bring-No-Ivory-Home-Campaign.aspx> | 2019 |
| 16 | 2014 | Give Peace to Elephants; Say No to Ivory Gifts Campaign | IFAW, USAID, Freeland Foundation | China | Chinese | <https://web.archive.org/web/20140302061511/www.ifaw.org/united-states/news/new-year-china-comes-new-campaign> | 2014, 2017 |
| 17 | 2014 | Last Days of Ivory | WildAid | Global | International | <http://www.lastdaysofivory.com/> | CWC/SE |
| 18 | 2014 | Ivory Free Campaign China | WildAid | China | Chinese | <https://wildaid.org/wildaid-launches-ivory-free-campaign/> | 2019 |
| 19 | 2014 | Green Collecting | TRAFFIC, Wen Wan Tian Xia | China | Chinese | <http://www.trafficchina.org/node/1983/> | CWC/SE |
| 20 | 2015 | Thailand Chor Chang campaign | WWF-Thailand | Thailand | Thai | <https://www.youtube.com/watch?v=PvnBmb0FT9Y> | 2019 |
| 21 | 2015 | I’m a Little Elephant | Humane Society International | Vietnam | Vietnamese | <https://iwt.challengefund.org.uk/documents/XXIWT006/24605/IWT006%20FR%20-%20edited.pdf> | 2014 |
| 22 | 2015 | China-Africa Wildlife Ambassadors initiative | IFAW | China | Chinese | <https://www.chinadaily.com.cn/2015-12/08/content_22654905.htm> | 2019 |
| 23 | 2015 | Rewrite their future campaign | WWF-HK, Geometry Global, Ogilvy Hong Kong | Hong Kong SAR | Chinese | <https://www.wwf.org.hk/en/?14480/Press-Release-WWF-Hong-Kong-calls-for-public-support-to-Rewrite-the-Future-of-African-ElephantsStop-the-trade-by-renaming-ivory-on-rewritetheirfuturewwforghk> | CWC/SE |
| 24 | 2016 | Elephant New Year Campaign | WildAid | China | Chinese | <https://www.adsoftheworld.com/campaigns/year-of-the-elephant> | CWC/SE |
| 25 | 2016 | Ivory Free Thailand Campaign | WildAid, WWF-Thailand, Save the Elephants, African Wildlife Foundation | Thailand | Thai | <https://www.youtube.com/watch?v=RxLHk6PVgNY> | CWC/SE |
| 26 | 2016 | Link your fingers to support elephants campaign | TRAFFIC, WWF-China | China | Chinese | <https://www.traffic.org/news/changing-consumer-choice-advice-a-few-clicks-away/> | CWC/SE |
| 27 | 2016 | The Ivory Game | Vulcan, Global Wildlife Program - GEF, Appian Way, Malaika Pictures, Terra Mater Film Studios | Global | International | <https://www.appsolutelydigital.com/WildLife/resources/Vulcan_Ivory_Game.pdf> | CWC/SE |
| 28 | 2017 | Ivory Free Vietnam Campaign | WildAid, CHANGE | Vietnam | Vietnamese | <https://wildaid.org/wildaid-and-change-launch-ivory-free-campaign-in-vietnam/> | CWC/SE |
| 29 | 2017 | I am #ivoryfree campaign Thailand | USAID-Asia-RDW, WildAid | Thailand | Thai | <https://www.usaidrdw.org/resources/reports/inbox/i_am_ivoryfree_report.pdf/view> | CWC/SE |
| 30 | 2018 | Wildlife Protection Law Campaign | USAID-Asia-RDW, IFAW | China | Chinese | <https://www.usaidrdw.org/campaigns/chinas-wildlife-protection-law> | CWC/SE |
| 31 | 2018 | Digital Deterrence Campaign | USAID-Asia-RDW | Thailand | Thai | <https://www.usaidrdw.org/campaigns/digital-deterrence-phases-1-and-2> | CWC/SE |
| 32 | 2018 | Beijing Zoo exhibition | African Wildlife Foundation | China | Chinese | <https://www.awf.org/news/working-china-build-investment-and-awareness-protects-african-wildlife> | CWC/SE |
| 33 | 2018 | Travel Ivory Free campaign | WWF-Thailand, WWF-China, WildAid, Tourism Authority of Thailand, Bangkok Art & Culture Center, Thailand Department of National Parks, Wildlife and Plant Conservation, NokScoot Airline, Coalition to End Wildlife Trafficking Online, WWF-Greater Mekong, WWF-Japan, WWF-Singapore, TRAFFIC, Wildlife Justice Commission, IFAW, National Geographic, WCS, WWF-HK | Thailand, China | Chinese, Thai | <https://wwf.panda.org/wwf_news/?336012/WWF-Launches-Travel-Ivory-Free-Campaign> | CWC/SE |
| 34 | 2018 | Kungfu Panda Fights Extinction campaign | WildAid, Zoological Park Association | Thailand | Thai | <https://wildaid.org/resources/kung-fu-panda-fights-extinction/> | CWC/SE |
| 35 | 2019 | Contraband Customs campaign | WildAid, WWF-China, TRAFFIC, National Forestry and Grassland Administration, China Anti-smuggling Bureau of the General Administration of Customs | China | Chinese | <https://wwf.panda.org/wwf_news/?341850/OnOne-YearAnniversaryofChinasIvoryBanNewCampaignTargetsTravelersAbroad> | CWC/SE |
| 36 | 2019 | Green Collection Campaign | TRAFFIC | China | Chinese | <https://cites.org/sites/default/files/eng/com/sc/74/E-SC74-34-A3-R1.pdf> | CWC/SE |
| 37 | 2019 | Elephants are Like Us | British Embassy | Thailand | Thai | <https://www.gov.uk/government/news/social-media-campaign-to-combat-illegal-ivory-trade-in-thailand-comes-to-an-end#:~:text=The%20'Elephants%20Are%20Like%20Us,the%20illegal%20trade%20in%20wildlife> | CWC/SE |
| 38 | 2019 | Be their Bodhisattva Campaign | WildAid, CHANGE, Dinosaur Agency | Vietnam | Vietnamese | <https://campaignsoftheworld.com/tv/wildaid-change-vietnam-be-their-bodhisattva/> | CWC/SE |
| 39 | 2019 | Make Vietnam Proud, Say No to Ivory campaign | TRAFFIC, British Embassy | Vietnam | Vietnamese | <https://www.traffic.org/site/assets/files/13112/uwa-traffic-cwt-2019-digest.pdf> | CWC/SE |
| 40 | 2019 | Hankograph campaign | WildAid, Tears of African Elephant | Japan | Japanese | <https://noivory.jp/hankograph/> | CWC/SE |
| 41 | 2019 | Demand Reduction Campaigns | TRAFFIC, WWF-Laos, Souphanouvong University | Lao PDR | Chinese, Laotian | <https://iwt.challengefund.org.uk/documents/XXIWT071/25031/IWT071%20FR%20-%20edited.pdf> | CWC/SE |
| 42 | 2019 | No to ivory souvenirs and gifts | USAID-Asia-RDW, Golden Triangle Asian Elephant Foundation | Thailand | Chinese, Thai | <https://www.usaidrdw.org/resources/reports/inbox/no-ivory-souvenirs-and-gifts_campaign-report.pdf/view> | CWC/SE |
| 43 | 2019 | Shanghai Zoo Exhibition | African Wildlife Foundation | China | Chinese | <https://www.awf.org/news/awf-partners-shanghai-zoo-showcase-endangered-african-wildlife-species> | CWC/SE |
| 44 | 2019 | Spiritual Beliefs Campaign | USAID-Asia-RDW, WildAid, Thailand Department of National Parks, Wildlife and Plant Conservation | Thailand | Thai | <https://www.usaidrdw.org/campaigns/a-good-life-is-free-of-killing> | CWC/SE |
| 45 | 2019 | Beautiful Without Ivory campaign | USAID-Asia-RDW, USAID Wildlife Asia | Thailand | Thai | <https://www.usaidrdw.org/campaigns/beautiful-without-ivory> | CWC/SE |
| 46 | 2020 | Ma Weidu Campaign | WWF-China, German Federal Ministry for Economic Cooperation and Development | China | Chinese | <https://www.wwf.or.th/en/?370596/Consumer-Demand-for-Ivory-Remains-in-Decline-Fifth-Annual-Survey-in-China-Finds> | CWC/SE |
| 47 | 2020 | Red on Nose | WildAid, Tears of African Elephant | Japan | Japanese | <https://noivory.jp/RedOnNose/> | CWC/SE |
| 48 | 2020 | Buy 1, Get 15 campaign | WildAid, CHANGE | Vietnam | Vietnamese | <https://changevn.org/en/change-wildaid-release-their-secret-weapon-to-get-their-important-message-across-to-the-vietnamese-people-the-buy-1-get-15-promotion/> | CWC/SE |
| 49 | 2020 | Wildlife Free Gifting Campaign | USAID Wildlife Asia, USAID-Asia-RDW, IFAW, We Marketing Group | China | Chinese | <https://www.usaidrdw.org/campaigns/wildlife-free-gifting> | CWC/SE |
| 50 | 2021 | Bring Home Memories Not Regrets campaign | WildAid, China Wildlife Conservation Association | China | Chinese | <https://wildaid.org/resources/bring-home-memories-not-regrets/> | CWC/SE |
| 51 | 2021 | Mercy is Power campaign | TRAFFIC | Thailand | Thai | [https://www.traffic.org/news/thais-urged-to-pledge-against-buying-and-owning-ivory-and-tiger-amulets-under-new-mercy-is-power-campaign](https://www.traffic.org/news/thais-urged-to-pledge-against-buying-and-owning-ivory-and-tiger-amulets-under-new-mercy-is-power-campaign/#:~:text='Mercy%20is%20Power'%20is%20a,from%20the%20Global%20Environment%20Facility.) | CWC/SE |
| 52 | 2022 | Our Shared World | IFAW | China | Chinese | <http://k.sina.com.cn/article_7517400647_1c0126e47059027kom.html> | CWC/SE |
| 53 | 2022 | Only Elephants Wear Ivory Best | WildAid, USAID-Asia-RDW, Thailand Department of National Parks, Wildlife and Plant Conservation | Thailand | Thai | <https://www.usaidrdw.org/campaigns/only-elephants-wear-ivory-best> | CWC/SE |
| 54 | 2022 | Say No to Shipping Illegal Wildlife Products | WildAid, China Wildlife Conservation Association, China Express Association | China | Chinese | <https://wildaid.org/wildaids-new-demand-reduction-campaign-targets-express-shipping/> | CWC/SE |
| 55 | 2022 | Wildlife Free Traveler Campaign | USAID-Asia-RDW, WildAid | Thailand | Chinese, Thai | <https://www.usaidrdw.org/campaigns/wildlife-free-traveler> | CWC/SE |
| **Interventions below (ID 56 – 75) were not included in analysis due to lack of available data.** | | | | | | | |
| 56 | 2008 | Raising Awareness of IWT Online | IFAW, Taobao, Ministry of Public Security of China, CITES Management Authority of China | China | Chinese | <https://cites.org/eng/news/world/19/3.php> | CWC/SE |
| 57 | 2011 | African Radio Campaign | TRAFFIC, China Wildlife Conservation Association, China Radio International | Africa (continent-wide) | Chinese | <https://www.traffic.org/news/chinese-in-africa-told-dont-buy-illegal-ivory/> | CWC/SE |
| 58 | 2013 | Alibaba Zero-Tolerance Campaign | Global Wildlife Program - GEF, Alibaba, GIZ | Global, China | International | <https://www.appsolutelydigital.com/WildLife/resources/Germany_Polifund.pdf> | CWC/SE |
| 59 | 2013 | Battle for the elephants | WCS, National Geographic | Global | International | <https://www.nationalgeographic.org/education/channel/battle-for-elephants/> | 2014, 2019 |
| 60 | 2013 | Ivory War Campaign | WildAid | China | Chinese | <https://www.youtube.com/watch?v=ccRAHhBoUgM> | 2014, 2019 |
| 61 | 2013 | Think Twice | IFAW | South Africa | International | <https://www.bizcommunity.com/Article/196/628/92816.html> | 2019 |
| 62 | 2014 | Board of Trustees Anti-Ivory Pledge | The Nature Conservancy | China | Chinese | <https://www.nature.org/media/annualreport/2014-annual-report.pdf> | 2014, 2019 |
| 63 | 2014 | Corporate ivory pledge led by Huang Nubo | WildAid | China | Chinese | <https://wildaid.org/chinas-top-business-leaders-say-no-to-ivory/> | 2014 |
| 64 | 2015 | Warlords of Ivory Documentary | National Geographic, Tusk, United for Wildlife | Global | International | <https://www.prnewswire.com/news-releases/prince-williams-ngo-tusk-discusses-terrorism-and-ivory-at-screening-of-warlords-of-ivory-documentary-300181876.html> | CWC/SE |
| 65 | 2015 | Tencent Penguin Loves Planet Earth | TRAFFIC, Tencent | China | Chinese | <https://www.traffic.org/news/traffic-and-tencent-sign-agreement-to-tackle-illicit-wildlife-trade-through-social-media-networks/> | CWC/SE |
| 66 | 2016 | Bonding with Giants | Humane Society International; Jane Goodall Institute; Rattle the Cage | Global | International | <https://www.rattlethecage.org/> | 2014 |
| 67 | 2017 | China-Uganda Initiative | WCS | Uganda | Chinese | <https://uganda.wcs.org/china-uganda-initiative.aspx> | 2019 |
| 68 | 2018 | Coalition to End Wildlife Trafficking Online | WWF, IFAW, TRAFFIC | China | Chinese | <https://www.endwildlifetraffickingonline.org/aboutthecoalition> | CWC/SE |
| 69 | 2019 | Stop Wildlife Crime National Awareness-raising Campaign | TRAFFIC, WWF-Laos | Lao PDR | Chinese | <https://iwt.challengefund.org.uk/documents/XXIWT071/25031/IWT071%20FR%20-%20edited.pdf> | CWC/SE |
| 70 | 2020 | Wildlife-Free E-Commerce Initiative | China Biodiversity Conservation and Green Development Foundation | China | Chinese | <http://www.cbcgdf.org/English/NewsShow/4997/12168.html> | CWC/SE |
| 71 | 2021 | Awareness-raising activities targeting Chinese and local Lao communities in Luang Namtha, Oudomxay | WWF-Laos | Lao PDR | Chinese | <https://iwt.challengefund.org.uk/project/XXIWT071/> | CWC/SE |
| 72 | 2021 | Losing Face campaign | IFAW | China | Chinese | <https://www.ifaw.org/uk/about/annual-report/2022/wildlife-crime> | CWC/SE |
| 73 | 2021 | Stop Wildlife Products Smuggling, Safeguard Biodiversity Customs Campaign | WildAid, TRAFFIC, WCS, IFAW, Anti-smuggling bureau of the General Administration of Customs (China) | China | Chinese | <https://wildaid.org/protect-biodiversity-by-saying-no-to-wildlife-smuggling/> | CWC/SE |
| 74 | 2022 | Protect Our Home Planet Campaign | Huya, China Wildlife Conservation Association, TRAFFIC, IFAW | China | Chinese | <https://www.traffic.org/news/level-up-for-tiger-protection-huya-launches-protect-our-home-planet-campaign-ahead-of-lunar-new-year/> | CWC/SE |
| 75 | 2023 | Reducing the Demand for Ivory Project | WWF-Vietnam, Vietnam National Administration of Tourism, Vietnam CITES Management Authority, GIZ | Vietnam | Vietnamese | <https://vietnamtourism.gov.vn/en/post/17656> | CWC/SE |
| ***Initial source(s) Key:** - 2014 = Sharif, V. (2014) Analysis of demand-side reduction initiatives. Stop Ivory, EPI and The Royal Foundation. Available at: <https://www.elephantprotectioninitiative.org/_files/ugd/f42bce_883d6a3ac9dd452586cf0a4d3c02b4da.pdf>  - 2017 = USAID Reducing Demand for Wildlife (2017) Summaries of Consumer Research, Campaign Evaluation Studies and other Literature relevant to Demand for Wildlife Parts and Products in China, Vietnam and Thailand. USAID Reducing Demand for Wildlife. Available at: <https://www.usaidrdw.org/resources/reports/inbox/usaid-wildlife-asia.pdf/view>  - 2019 = Veríssimo, D. and Wan, A. K. Y. (2019) ‘Characterizing efforts to reduce consumer demand for wildlife products’, Conservation biology: the journal of the Society for Conservation Biology. John Wiley & Sons, Ltd, 33(3), pp. 623–633. Available at: <https://doi.org/10.1111/cobi.13227>  - CWC/SE = Search strategy starting point of the Change Wildlife Consumer Resource Library. Available at: <https://www.changewildlifeconsumers.org/resource/> - followed by relevant Search Engine sampling. | | | | | | | |

# S2. Data Collection & Analysis

### *Data Collection: Literature Review*

The lead author conducted the review, as she is a native English speaker and speaks Mandarin Chinese to a professional fluency, with experience reading and writing in both simplified and traditional characters for 15+ years.

The implementation organizations’ websites and resource directory web pages were the most frequent sources of further information. As much detail as possible was recorded for each intervention to enable sufficient cross referencing to remove duplicates, which frequently occurred when partner organizations used modified names or phrases to refer to the same intervention.

### *Analysis: NSMC Benchmark Criteria*

The NSMC Benchmark Criteria are not mutually exclusive, and they are most successful when adhered to holistically throughout the design and implementation of an intervention aiming to influence human behavior. By referring to these criteria as the conceptual framework for effective behavior change, we can use an intervention’s integration of these principles and the quality of this integration, or lack thereof, to understand each intervention’s potential to change behaviour. We also considered directly related socioeconomic, political, and cultural phenomena. For instance, relevant anti-corruption or political movements, domestic ivory bans, ivory stockpile destruction events, economic recessions, and pandemics and their effects were noted.

We recommend reading the guidelines for the NSMC Benchmark Criteria available at: <https://www.thensmc.com/content/nsmc-benchmark-criteria-0>, where supporting ‘how-to-use’ resources and case studies are available to help with application for designing social marketing interventions.

### *Analysis: Social Marketing Indicator (SMI)*

Description of the SMI *clusters* and *steps* based on Wettstein and Suggs (2016) is provided below to help elucidate how they provide the critical framework for achieving successful social marketing intervention. To answer the SMI questions for each step rigorously, and with the most relevance for ivory demand management, we adapted the original SMI tool from binary *yes* or *no* answers, to include an additional third answer of *partial* in lieu of some but not clear-cut evidence that a design or implementation choice suggested that the step had been completed. In cases where no evidence was available at all to indicate step completion, the answer of *no* was chosen. Where evidence clearly demonstrated the integration of the social marketing principles underpinning a step, the answer of *yes* was chosen. These qualitatively based SMI indicators were converted into a simple scoring system where *no* = 0, *partial* = 1, and *yes* = 2 for the purposes of visualizing the results. *Process integrity* refers to the essential steps that must be carried out at some point during the design or implementation of an intervention. *Process quality* subsequently refers to the undertaking of these steps, questioning their quality based on if they were customer-focused and evidence-based (Wettstein and Suggs, 2016).

1. *Scope and Direction:* According to Wettstein and Suggs (2016), there are four steps required in the Scope and Direction cluster. First, for every social marketing intervention, there must be a guiding, clearly defined *purpose*. Second, this will enable specific *target* groups to be decided based on research evidence. Third, once the target group(s) are defined, intervention *goals* need to be established. Fourth, goals should be defined in terms of target audience behavior to allow specific *objectives* to guide the attainment of the goals to fulfil the purpose of the intervention (Wettstein and Suggs, 2016).
2. *Exchange and Competition:* According to Wettstein and Suggs (2016), in the Exchange and Competition cluster of the SMI, the exchange strategy (i.e., what does the intervention offer in exchange for the target group performing the desired behavior) should be defined by three steps, that begin with establishing a behavioral *theory*. This should be chosen based on research evidence, which will result in delivering the second step of considering the *exchange* of benefits for the target group, outweighing the costs based on the behavior change incited. Once defined, research should be conducted for the third step, which considers the factors that threaten achieving the behavior, i.e., *competition*, along with finding counteracting measures (Wettstein and Suggs, 2016).
3. *Marketing Mix:* According to Wettstein and Suggs (2016), the Marketing Mix cluster should align with McCarthy’s (1960) traditional principles of marketing, known as the “Four Ps” (Yudelson, 1999)^[[1]](#footnote-1)^. These include *Promotion* i.e., of an actual behavior, *Product* i.e, the benefits of the behavior, *Price* i.e., incentives and/or disincentives, and *Place* i.e., where, when and how the promotion is conducted. For robust implementation, these choices should be based on research and must be pretested with the target audience (Wettstein and Suggs, 2016).
4. *Monitoring and Evaluation:* According to Wettstein and Suggs (2016) to complete the two steps of the Monitoring and Evaluation cluster, once the intervention has been implemented, first, it must be *monitored* in terms of process and outcomes, and adaptations should be made based on monitoring results, if necessary. Second, once implementation is finished, then an *evaluation* must take place in terms of outcomes (i.e., behaviors) and impacts (i.e., problem resolution) (Wettstein and Suggs, 2016).

## S3. Interview Guidelines and Privacy Notice

### Interview overview for ivory demand reduction practitioners

These will be semi-structured interviews. All questions are open-ended to allow for deep conversation around the topics under question. Questions will not be specifically asked if the answer has been provided in response to other questions. Additionally, follow-up questions within the theme of the topics covered here will be asked if necessary for further information.

### Organisational question topics

1. Role(s) of the interviewee in the organisation (present/past)
2. Past demand reduction activities involved in ivory
3. Specific training in Social Marketing or Social Behaviour Change Communications practices
4. Engagement in social marketing associations (e.g. iSMA, ESMA)
5. Engagement in national or international meetings to tackle illegal wildlife trade via demand reduction
6. Broader / other experiences relevant to the development of demand reduction interventions

### Questions about identified interventions

Each interviewee will have identified specific interventions they have been involved with in the initial Screening Questionnaire filled out before their interview. Based on these results, a list of interventions will be curated for each interviewee and questions will be tailored to these specific interventions.

1. Was an intervention purpose defined?
   1. Was the intervention purpose linked to a specific problem?
2. Did the campaign set goals for target behaviours?
   1. What was the primary target behaviour?
   2. Were general goals defined?
      1. Were goals focused on behaviour?
      2. Was the choice of goals based on evidence?
      3. Were specific goals defined?
3. Selection of the target audience
   1. Was at least one target group defined?
   2. Was the choice of target groups based on evidence?
4. Were behavioural theories used in campaign design?
   1. Which theory was used to understand how the target group(s) can be influenced?
      1. Why was this theory used? Was this choice based on evidence?
      2. If not, why was a theory-based approach not used?
5. Did you do audience research?
   1. Were key factors motivating the consumer captured?
      1. Were actionable insights generated from the capture of consumer insights?
      2. Was the campaign material pretested with the target group?
6. Were the ‘benefits’ and ‘costs’ of the desired behaviour framed and communicated?
   1. Were costs for the target group(s) considered?
      1. Was the identification of costs for the target group(s) based on evidence?
   2. Were benefits for the target group(s) considered?
      1. Was the identification of benefits for the target group(s) based on evidence?
      2. Were the benefits of the recommended behaviour promoted?
   3. Was an exchange of benefits for the target audience proposed?
7. Was the role of ‘competition’ considered (e.g. for the potential target audience’s time, attention and inclination to act)?
   1. How was the role of competition assessed, which theories or methods did you use?
   2. Were measures to counteract the competition considered?
      1. Were the measures taken to counteract the competition based on evidence?
8. Were incentives or disincentives considered and what were they?
   1. Why was this decision made to incentivise or disincentivise the target audience?
9. Was segmentation of the target audience into groups considered, and how?
   1. Were these groups identified based on evidence?
   2. How were these identified groups targeted?
   3. Was positioning considered for the target groups? E.g. brand positioning of the organisation, or the positioning of the specific campaign?
10. Was a mix of marketing methods considered? (This is also known as the “4Ps” for Product, Price, Place, and Promotion. This relates to creating the right product, promoted at the right price and delivered in the right place for the target audience).
    1. What elements of the marketing mix elements were most important in this campaign and why?
11. Was there at least one clearly stated message?
    1. Was the message distributed through different channels?
12. Was the campaign monitored in terms of behavioural outcome(s)?
    1. Were campaign goals (e.g. knowledge, awareness) measured?
13. Was adaptation of campaign elements considered?
    1. When was this considered? Was it proactive or reactive? E.g. was their consideration or action of adaptation to the campaign during a pilot, or during the campaign rollout?
14. Was evaluation done in terms of behaviours? E.g. relating to the outcome of the campaign
15. Was the evaluation done in terms of problem resolution? E.g. relating to the impact of the campaign

**Privacy Notice**

**Project title: Assessing ivory demand reduction interventions using social marketing**

The information you provide in these interviews will be used by University of York PhD student

Molly Brown, funded by the Leverhulme Trust through the Leverhulme Centre for Anthropocene

Biodiversity (LCAB). The information provided will be used in a study to assess ivory demand

reduction interventions using a social marketing approach.

The purpose of this research is to collect information about specific, identified ivory demand

reduction interventions, particularly where data is lacking in the published literature and to gain

a better holistic understanding of the experiences of working on ivory demand reduction

interventions.

The University of York processes personal data for research purposes under Article 6 (1) (e) of

the GDPR (General Data Protection Regulation) which allows: *Processing necessary for the*

*performance of a task carried out in the public interest.*

The research will only be undertaken where ethical approval has been obtained by the

University of York Biology Ethics Committee, where there is a clear public interest and where

appropriate safeguards have been put in place to protect data.

In line with ethical expectations and in order to comply with the common law duty of

confidentiality, we will seek your consent to participate where appropriate. This consent will not,

however, be our legal basis for processing your data under the GDPR.

Data (e.g. transcripts of interviews) will be shared with Molly Brown and her PhD supervisors

Prof Colin Beale and Prof Victoria Wells, who sit within the University of York’s Biology

Department and School for Business and Society, respectively.

Participants can withdraw from this project, including withdrawal of information provided in part

or in full during interviews, up to 3 months after the interview has been conducted. After this

point, the interview data will be incorporated into the study analyses and will not be able to be

withdrawn.

If you have any questions about this privacy notice or concerns about how your data is being

processed, please contact Molly Brown at [molly.brown@york.ac.uk](mailto:molly.brown@york.ac.uk) or the University of York’s

Data Protection Officer at [dataprotection@york.ac.uk](mailto:dataprotection@york.ac.uk). More information can also be found [here](https://www.york.ac.uk/records-management/dp/your-info/generalprivacynotice/).

**S4. Results**

Intervention overview: Interventions varied widely, but typically an intervention consisted of different physical and digital components, mass media communication strategies, multiple roll-outs over time, multiple and varied celebrity endorsements, public service announcements (PSAs), and pledges.

Seven interventions remained void of four or more Benchmarks from 2018 to 2022, including *Beijing Zoo Exhibition* (2018), *Kungfu Panda Fights Extinction* (2018), *Elephants are Like Us* (2019), *Make Vietnam Proud, Say No to Ivory* (2019), *Hankograph* (2019), *Shanghai Zoo Exhibition* (2019), and *Red on Nose* (2020). Of the nine interventions that integrated both monitoring and evaluation to some extent, six were run by USAID-WA, and the other three: *Travel Ivory Free* (Thailand and China, 2018), *Demand Reduction Campaigns* (Lao PDR, 2019), and *Ma Weidu Campaign* (China, 2020), were run by WWF-Thailand, WWF-Laos, and WWF-China, respectively, collaborating with government partners, universities, and in two of these instances with TRAFFIC.

We provide ten examples of interventions and social marketing principles integration below:

1. *Digital Deterrence* (2018) - Audience segments were based on their online search history of ivory-related searches. Each group was presented with different messages based on the likelihood of their search leading to a desire to purchase ivory. The variation in materials shown to the audience segments demonstrates the consideration for different barriers and facilitators to ivory consumption for different groups. A tripartite approach to messenger, message, and marketing mix was used to reach the audience segments appropriately. Whereas, those less likely to be ivory consumers, were shown celebrity-endorsed messages that tried to reduce associations between ivory and social and aesthetic values. This group was comparatively directed to a pledging page to commit to stopping ivory purchases. This approach establishes different means of influencing the specific behavior of each audience segment based on the unique values they are likely to hold towards ivory.
2. We found less clear segmentation underpinning other interventions such as *Bring Home Memories Not Regrets* (2021) and *Wildlife Free Traveler* (2022). In these examples, Chinese tourists were the primary target audience. However, they were broadly targeted at airports and train stations using mass-media and PSAs without further targeting to segment the proportion of tourists more likely to purchase ivory abroad. We found no specific strategy in the interventions such as a relevant messenger, place, or promotional activity that could indicate an additional level of segmentation had been considered. This undermines the quality of the steps achievable across the SMI. These interventions were less likely to effect ivory consumption behaviors, given their broad approach to segmenting their audience.
3. *Buy 1, Get 15* (2020) - In further messaging, a similar sentiment was repeated with a familial focus, e.g., “15 years in prison can make a person’s youth and life exhausted, ...can make children lose their fathers, ...can make a good wife bear more burdens”. This repetition, with unique family values, could help audiences align their own values with the intervention, providing more chances for audience attachment to the messaging and greater likelihood of affecting their behavior through more precise value targeting. Concepts related to Competition were also well considered in the intervention *Buy 1, Get 15 campaign* (2020). For example, the primary message relays that “no gift is worth 15 years in prison”, clarifying for the audience the likely loss of freedom if they were to buy ivory as a gift. This shows that the social costs to the consumer were considered in the design of the intervention.
4. *Wildlife Free Gifting* (2020) - Here, principles of exchange were found in the design choice to focus on helping the audience choose an appropriate gift as the focal messaging point. This shows consideration for the circumstances under which a consumer may feel obliged to buy an ivory product, and at what social cost. The intervention highlighted the social and punitive perceived versus actual costs of gifting ivory. We found that competition principles were effectively integrated in *Wildlife Free Traveler* (2022). This intervention targeted the competing factors affecting Chinese tourists’ spontaneous consumption of ivory, which translated into the intervention purposely increasing the perceived risk of ivory as a souvenir or gift. These choices were based upon the findings that “those who traveled abroad would tend to buy it, not because they travel abroad to buy ivory, but because it happened that they see it, they like it, and then they buy it as souvenirs and gifts” (P2).
5. *Contraband Customs Campaign* (China, 2019) and *Mercy is Power* (Thailand, 2021) - Evaluation was heavily focused on engagement i.e., the number of people reached by the promotional materials, and did not assess behavioral outcomes or problem resolution. This is likely to due to the lack of baseline and monitoring data collected throughout the life cycle of the interventions.
6. Pretesting – Piloting evidence provided rationale for intervention adaptation based on insights from consumer focus groups and interviews for USAID-WA/RDW. P2 explained that “pretesting was a very necessary step to all the campaigns... so we had very systematic testing... followed by approval by the government”. Therefore, pretesting not only uncovered weaknesses in need of address to ensure audiences were connected with appropriately, but also gave logistical opportunity to ensure buy-in from government bodies as “obviously there’s going to be approval at every single stage from the government” (P2). The requirement for government support for intervention roll-out is a critical component enabling the continuation and macro-level support required for follow-up interventions.
7. Marketing Mix - “Initially, we wanted really to display ads in the malls, in this amulet market, but we were not allowed to because of course, it is contrary to the interests of the mall” (P5). This highlights how *Promotion* was considered within the design of the intervention, but the most appropriate *Place* to target the consumer group desired could not be achieved due to the wider problems it may cause.
8. Evaluation - Interviewees noted their frustration in not knowing “if you reach these people, whether they actually were engaged, whether they saw the video or whether they really watched the video or they saw the billboard” (P2).
9. *Spiritual Beliefs* (2019) - In this instance, monks were encouraged to sign a commitment poster to pledge their active support to help prevent wildlife crime and reduce consumer demand for wildlife. The pledge here had a broader focus but still indicates a positioning of the spiritual leaders in the ivory landscape as important messengers of change, and by pledging, reminds the monks of their capacity to lead by example for their followers.
10. *Green Collecting* (2014/2019) - Here, in 2014 a pledge was used to encourage master collectors to reduce ivory consumption and guide the new trend of green collection, which was repeated with different master carvers and collectors in the follow up intervention in 2019.

## S5. Results - NSMC Benchmark Criteria Analysis


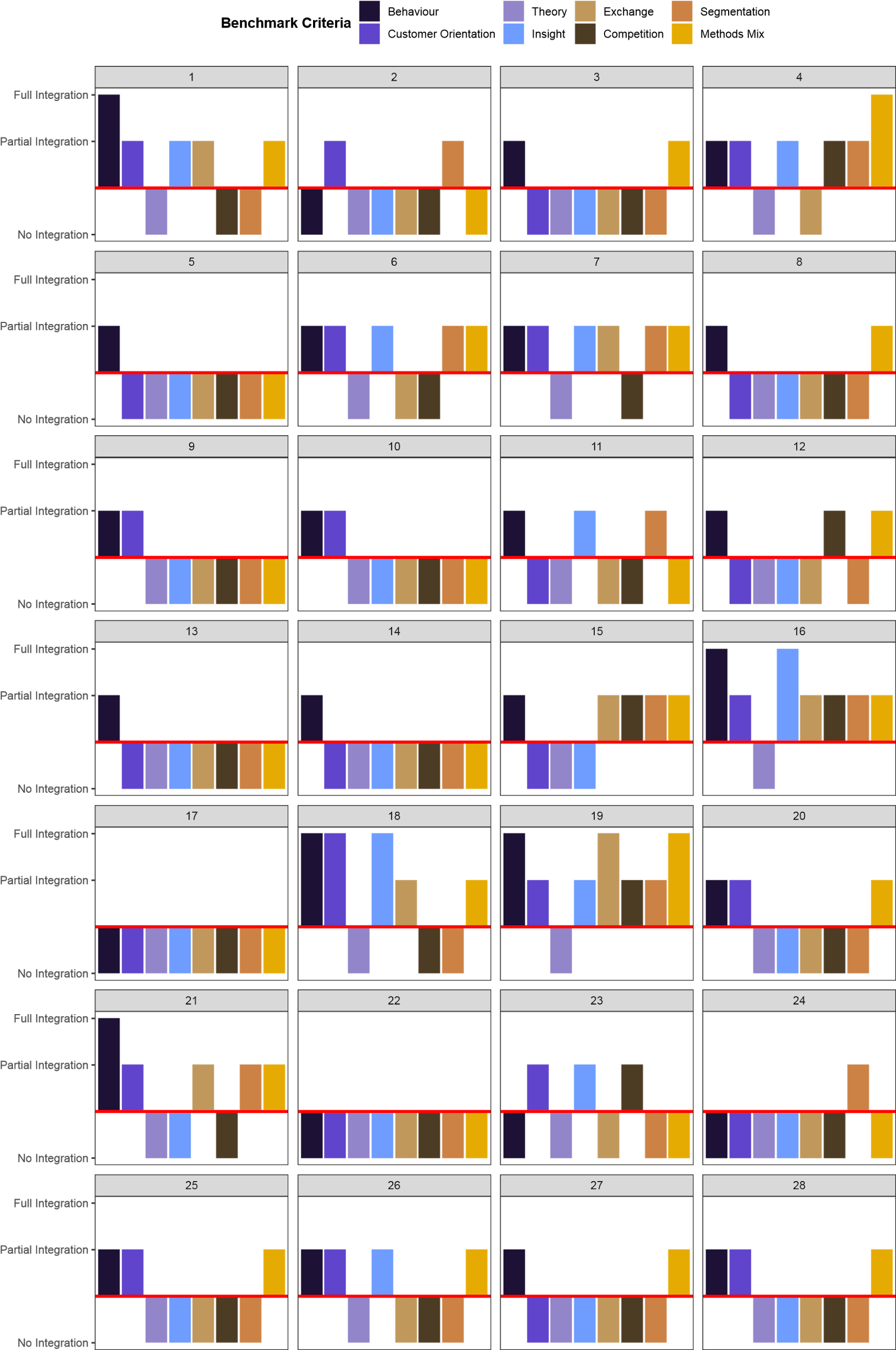


Benchmark Criteria Integration


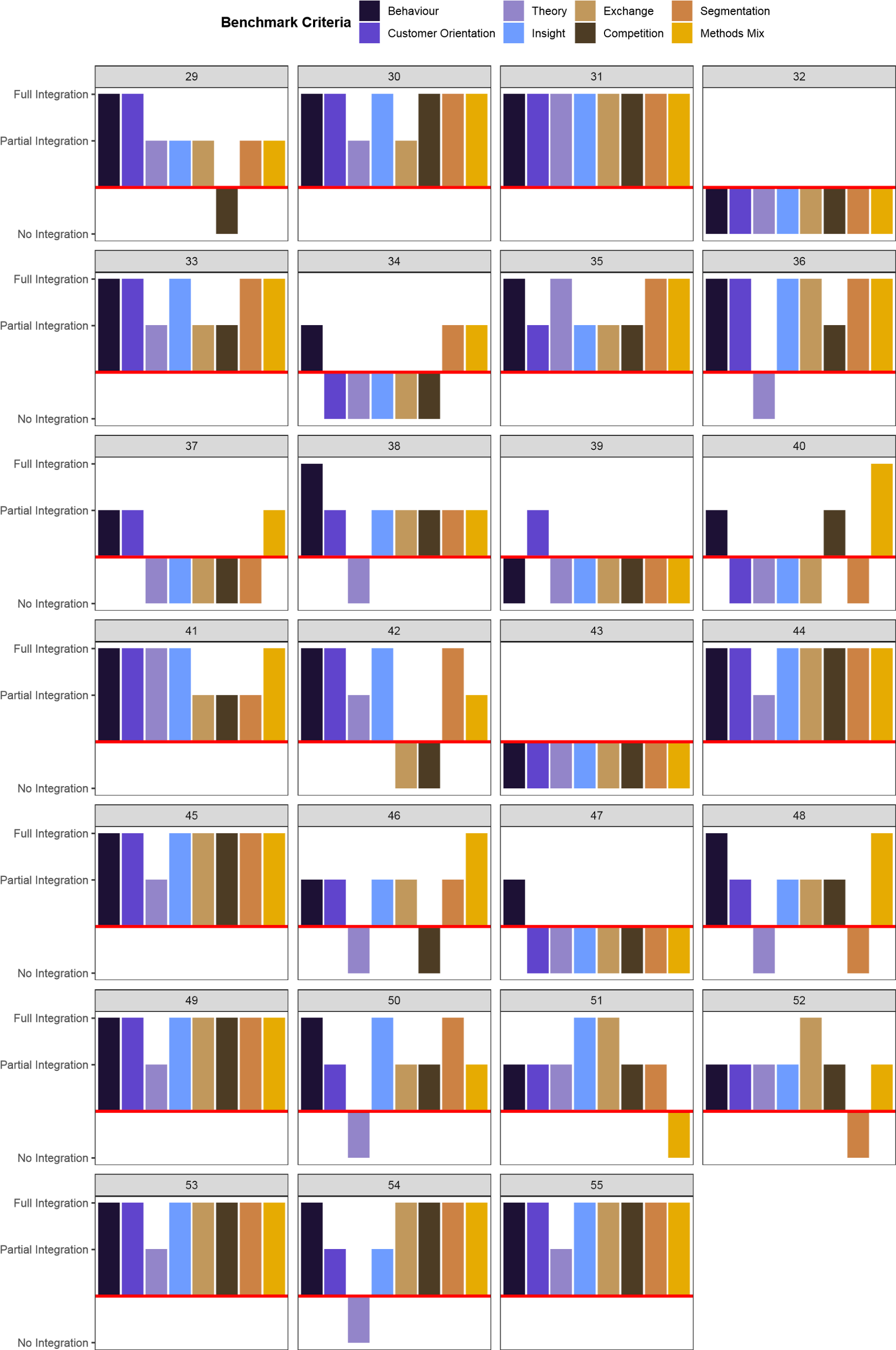


Benchmark Criteria Integration

## S6. Results - Social Marketing Indicator (SMI) Analysis


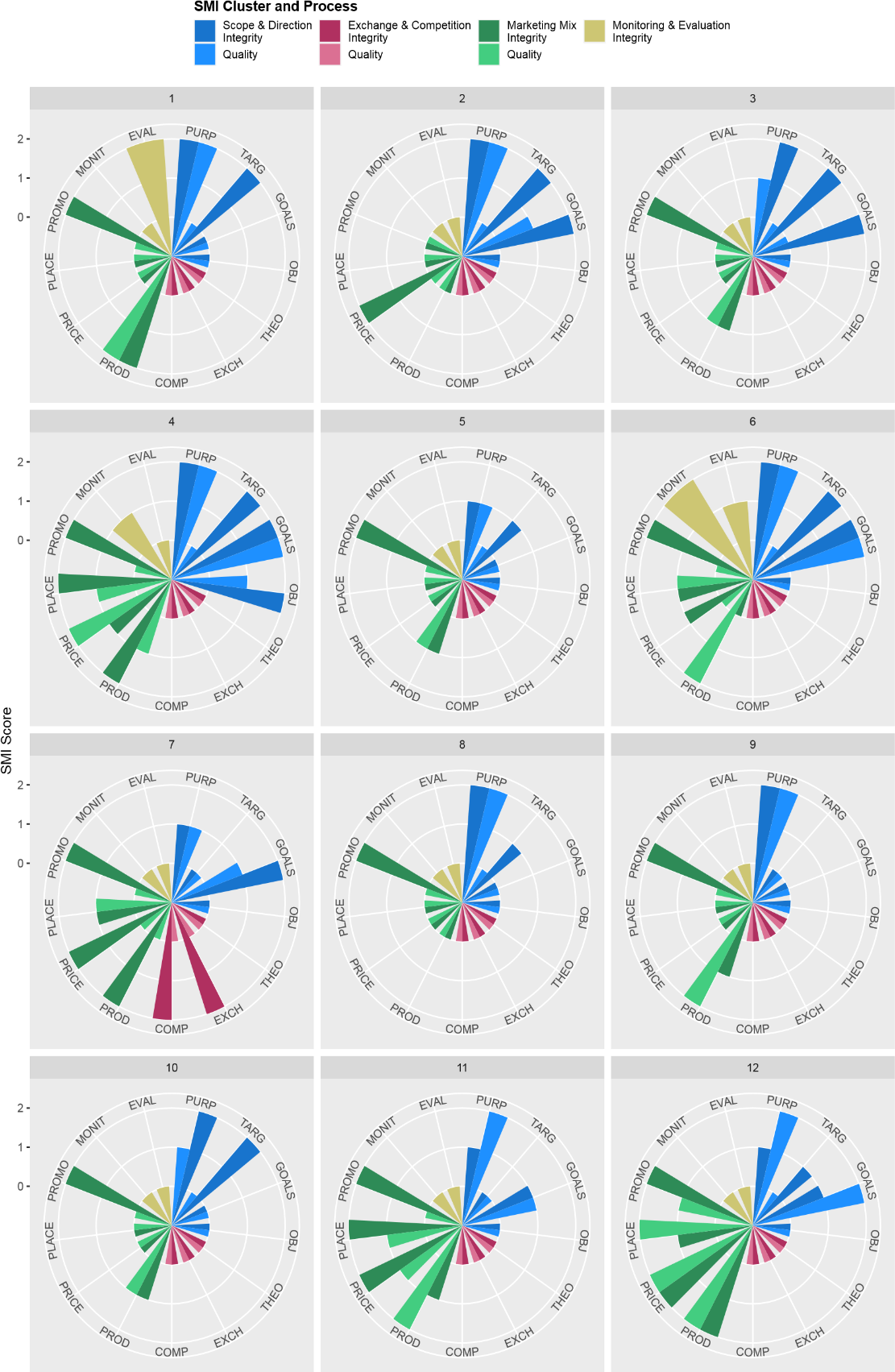


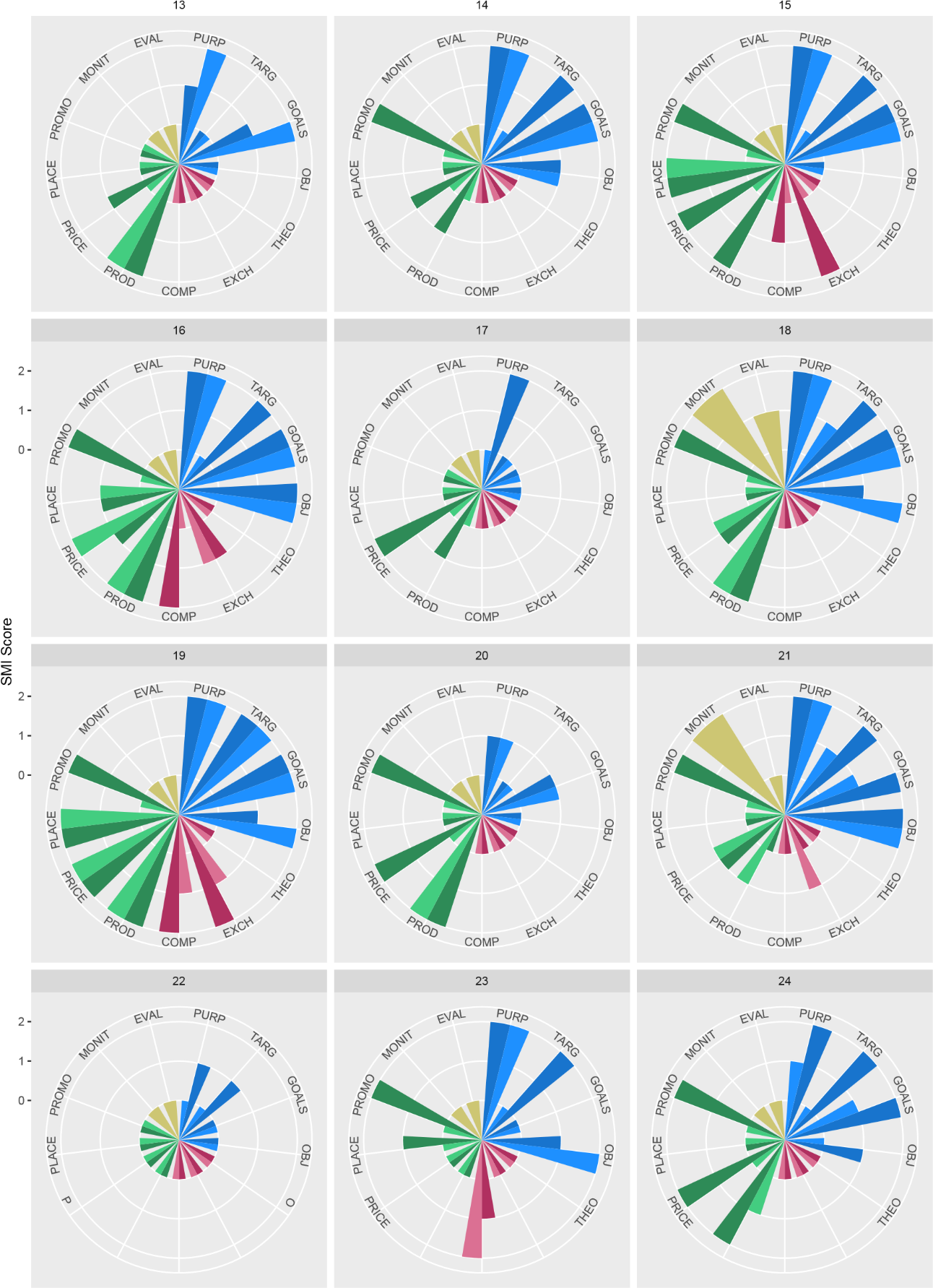


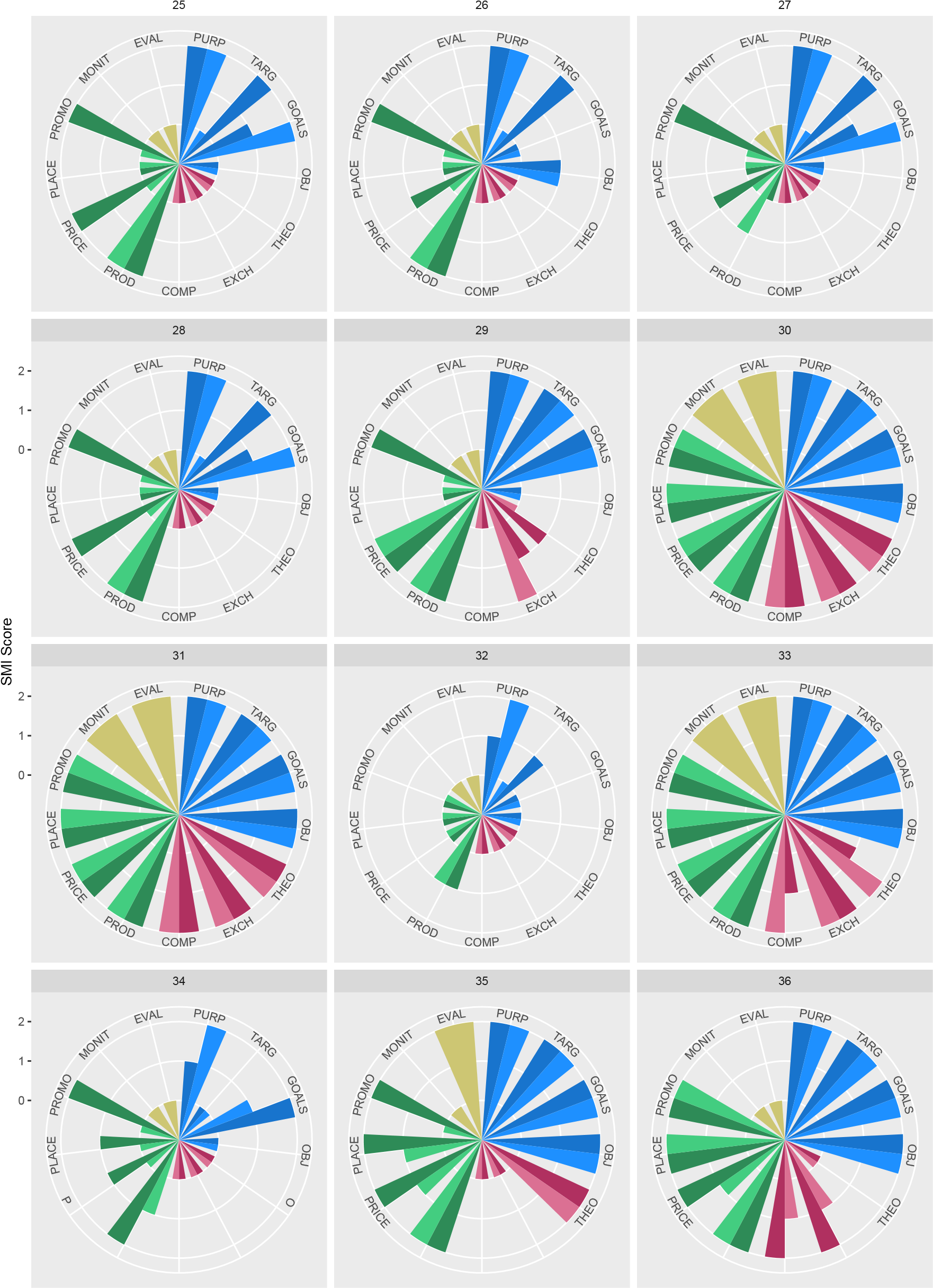


#
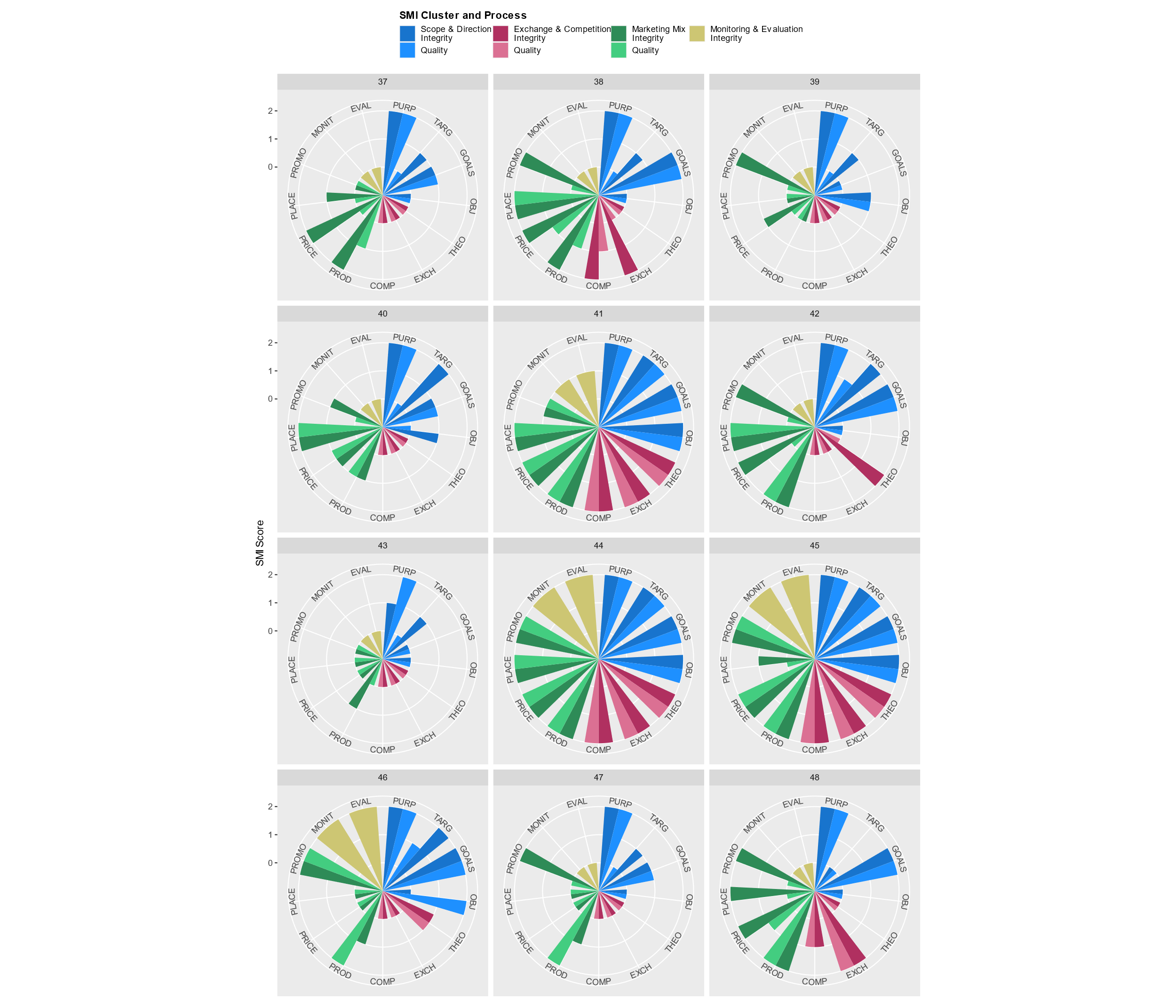

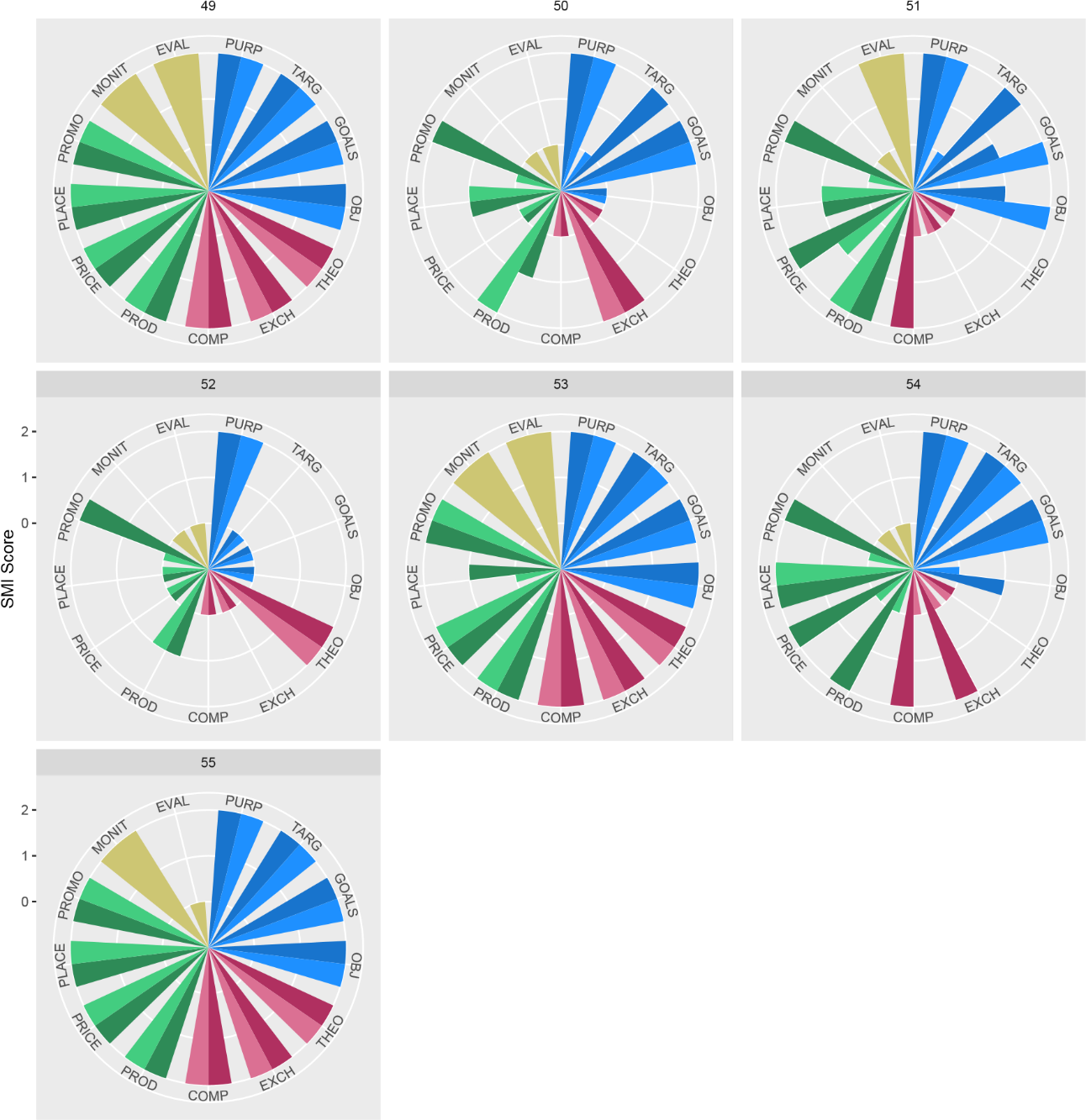


1. Yudelson, J. (1999). Adapting Mccarthy’s Four P’s for the twenty-first century. *Journal of Marketing Education*, 21(1):60–67. [↑](#footnote-ref-1)
